# A Goal-Directed Trajectory Planning Using Active Inference in UAV-Assisted Wireless Networks

**DOI:** 10.3390/s23156873

**Published:** 2023-08-02

**Authors:** Ali Krayani, Khalid Khan, Lucio Marcenaro, Mario Marchese, Carlo Regazzoni

**Affiliations:** 1 Department of Electrical, Electronic, Telecommunications Engineering and Naval Architecture, University of Genoa, 16145 Genoa, Italy; khalid.khan@edu.unige.it (K.K.); lucio.marcenaro@unige.it (L.M.); mario.marchese@unige.it (M.M.); carlo.regazzoni@unige.it (C.R.); 2 Italian National Inter-University Consortium for Telecommunications (CNIT), 43124 Parma, Italy

**Keywords:** UAVs, wireless networks, trajectory design, AI-enabled radios, active inference, world models, traveling salesman problem

## Abstract

Deploying unmanned aerial vehicles (UAVs) as aerial base stations is an exceptional approach to reinforce terrestrial infrastructure owing to their remarkable flexibility and superior agility. However, it is essential to design their flight trajectory effectively to make the most of UAV-assisted wireless communications. This paper presents a novel method for improving wireless connectivity between UAVs and terrestrial users through effective path planning. This is achieved by developing a goal-directed trajectory planning method using active inference. First, we create a global dictionary using traveling salesman problem with profits (TSPWP) instances executed on various training examples. This dictionary represents the world model and contains letters representing available hotspots, tokens representing local paths, and words depicting complete trajectories and hotspot order. By using this world model, the UAV can understand the TSPWP’s decision-making grammar and how to use the available letters to form tokens and words at various levels of abstraction and time scales. With this knowledge, the UAV can assess encountered situations and deduce optimal routes based on the belief encoded in the world model. Our proposed method outperforms traditional Q-learning by providing fast, stable, and reliable solutions with good generalization ability.

## 1. Introduction

In recent years, there has been a significant amount of research interest in unmanned aerial vehicles (UAVs) due to their impressive features, such as their maneuverability, ease of positioning, versatility, and the high likelihood of line-of-sight (LoS) air-to-ground connections [1,2]. UAVs are feasibly exploited to alleviate a wide range of challenges in commercial and civilian sectors [3,4]. It is expected that forthcoming wireless communication networks will need to provide exceptional service to meet the demands of users. This presents difficulties for traditional terrestrial-based communication systems, particularly in hotspot areas with high traffic [5,6,7]. UAVs have the potential to serve as flying base stations, providing support to the land-based communication infrastructure without the need for costly network construction [8]. In addition, their ability to be easily relocated makes them particularly highly beneficial in the aftermath of natural disasters [9,10]. UAVs can also be deployed as intermediaries between ground-based terminals, improving transmission link performance and enhancing reliability, security, coverage, and throughput [11,12]. As such, UAV-assisted communications are becoming increasingly vital in developing future wireless systems [13,14,15,16,17].

UAV-aided wireless communications possess a distinct advantage owing to the controllable maneuverability of UAVs, which allows for flexible trajectories. This added degree of freedom significantly boosts the system’s performance. Therefore, optimizing the UAV’s trajectory is an indispensable area of focus in this field, as it is paramount to exploit the potential of UAV-assisted wireless communications fully [18]. Several studies have looked into improving system performance through trajectory design. One study, for example, optimized the trajectory of a UAV to gather received signal strength measurements efficiently and improve the accuracy of spectrum cartography [19]. Another study proposed a method for planning the trajectory of a UAV to provide emergency data uploading for large-scale dynamic networks [20]. Multi-hop relay UAV trajectory planning is also crucial in UAV swarm networks [21]. Joint optimization of the UAV’s trajectory and user association was suggested in [22] to maximize total throughput and energy efficiency. Another study examined joint UAV trajectory design and time allocation for aerial data collection in NOMA-IoT networks [23]. In a cluster-based IoT network, joint optimization of the UAV’s hovering points and trajectory was studied to achieve minimal age-of-information data collection [24]. Autonomous trajectory planning solutions were proposed in [25] to enable UAVs to navigate complex environments without GPS while fulfilling real-time requirements. Lastly, the trajectory of a UAV was optimized in [26] to minimize propulsion energy and ensure the required sensing resolutions for cellular-aided radar sensing.

Traditional methods rely on optimization mathematical models that require precise information about the system, including the number of users in different areas and network parameters when designing a UAV trajectory. However, this approach may not be feasible in real-world situations due to the constantly changing environment and limited battery life, making it difficult to solve these problems using traditional techniques [27]. On the other hand, artificial intelligence (AI) techniques, such as machine learning (ML) and reinforcement learning (RL), have proven to be effective in addressing challenges related to sequential decision making. By equipping UAVs with AI capabilities (AI-enabled UAVs), they can attain a remarkable level of self-awareness, transforming wireless communications [28]. With AI, UAVs can effectively comprehend the radio environment by discerning and segregating the explanatory factors that are concealed in low-level sensory signals [29]. However, most ML and RL methods are not capable of adjusting to new situations that were not included in their initial training. This limitation in generalizing requires extensive retraining efforts, which can pose challenges for real-time prediction and decision making [30].

When AI-enabled agents sense and interact with their environment, they struggle with structuring the knowledge they gather and making logical decisions based on it. One way to address this is through knowledge representation and reasoning techniques inspired by human problem-solving to handle complex tasks effectively [31]. Causal probabilistic graphical models are a prime example of such techniques, which are highly effective in capturing the hidden patterns in sensory data obtained from the environment. These models also provide a seamless way to integrate sensory data from various sources [32]. By statistically structuring the data, they can describe different levels of abstraction that can be applied across different domains. For instance, when learning a language, one must learn how sounds form words, how words form sentences, and how grammar characterizes a language. At every level, the learning process requires making probabilistic inferences within a structured hypothesis space. Dealing with uncertainty is a common challenge in AI and decision making, as many real-world problems have incomplete or ambiguous information. Probabilistic representation is an effective technique that leverages probability theory to model and reason with uncertainty, enabling AI agents to make better decisions and operate more efficiently [33].

Active inference is a mathematical framework that helps us understand how living organisms interact with their environment [34]. It provides a unified approach to modeling perception, learning, and decision making, aiming to maximize Bayesian model evidence or minimize free energy [35]. Free energy is a crucial concept that empowers agents to systematically assess multiple hypotheses concerning behaviors that can effectively achieve their desired outcomes. Moreover, active inference governs our expectations of the world around us. Specifically, it posits that our brains utilize statistical models to interpret sensory information [36]. By using active inference, we can modify our sensory input to conform to our preconceived notions of the world and rectify any inconsistencies between our expectations and reality. Probabilistic graphical models are used to represent active inference models because they provide a clear visual representation of the model’s computational structure and how belief updates can be achieved through message-passing algorithms [37].

Motivated by the previous discussion, we propose a goal-directed trajectory design framework for UAV-assisted wireless networks based on active inference. The proposed approach involves two key computational units. The first unit meticulously analyzes the statistical structure of sensory signals and creates a world model to gain a comprehensive understanding of the environment. World models are a significant aspect of generative AI. They play a pivotal role in the development of intelligent systems. Like humans, AI agents acquire a world model by processing sensorimotor data through interactions with their environment, which serves as a simulator in their brains [38]. The second is the decision-making unit seeking to perform actions minimizing a cost function and generating preferred outcomes. The two components are linked by an active inference process. To create the world model, the UAV was trained to complete various flight missions with different realizations (such as the locations of hotspots and users’ access requests) using the conventional traveling salesman problem with profit (TSPWP) [39] with the 2-OPT local search algorithm in an offline manner. The TSPWP instances (trajectories) were turned into graphs and used to build a global dictionary with two sub-dictionaries. The first sub-dictionary represents the hotspots the UAV needs to serve and their order of travel. By contrast, the second sub-dictionary shows the trajectories to follow between two adjacent nodes. The global dictionary consists of letters at multiple levels, tokens, and words. The world model is created by coupling the two sub-dictionaries, constructing a detailed representation of the environment at different hierarchical levels and time scales. The world model is structured in a coupled multi-scale generalized dynamic Bayesian network (C-MGDBN). This model builds upon the single-scale GDBN, which is a statistical model that explains how hidden states drive time series observations. However, unlike the conventional GDBN [40,41,42], which can only model single-scale data, our enhanced GDBN representation can encode the dynamic rules that generate observations at different temporal resolutions, making it far more versatile than traditional GDBNs. With this superior model, we can simultaneously model a UAV’s behavior at different time scales. The decision-making unit relies on active inference to select actions based on the current state of the environment as inferred from the world model. The proposed framework explains how UAVs navigate their surroundings with a goal in mind, choosing actions that minimize unexpected or unusual observations (abnormalities), which are measured by how much they deviate from the expected goal.

The main contributions of this paper can be summarized as follows:

- We developed a global dictionary during training to discover the TSPWP’s best strategy for solving different realizations. The dictionary comprises letters representing the available hotspots, tokens representing local paths, and words depicting the complete trajectories and order of hotspots. By studying the dictionary, we can comprehend the decision maker’s grammar (i.e., the TSPWP strategy) and how it uses the available letters to form tokens and words.
- We have designed a novel hierarchical representation structuring the acquired knowledge (the global dictionary) in a C-MGDBN to accurately depict the properties of the TSPWP graphs at various levels of abstraction and time scales.
- We tested the proposed method on different scenarios with varying hotspots. Our method outperformed traditional Q-learning by providing fast, stable, and reliable solutions with good generalization ability.

The remainder of the paper is organized as follows. The literature review is presented in Section 2. The system model and problem formulation are presented in Section 3. The proposed goal-directed trajectory design method is explained in Section 4. Section 5 is dedicated to the numerical results and discussion, and finally Section 6 concludes this paper by highlighting future directions.

Notations: Throughout the paper, capital italic letters denote constants, lowercase bold letters denote vectors, and capital boldface letters denote matrices. The shorthand N(μ,Σ) is used to denote a Gaussian distribution with mean μ and covariance Σ. If X represents a matrix, the element in its *i*th row and *j*th column is denoted by $x_{ij}$, and its *i*th row vector is represented by $x_{i}$.

## 2. Literature Review

**Figure 1:**
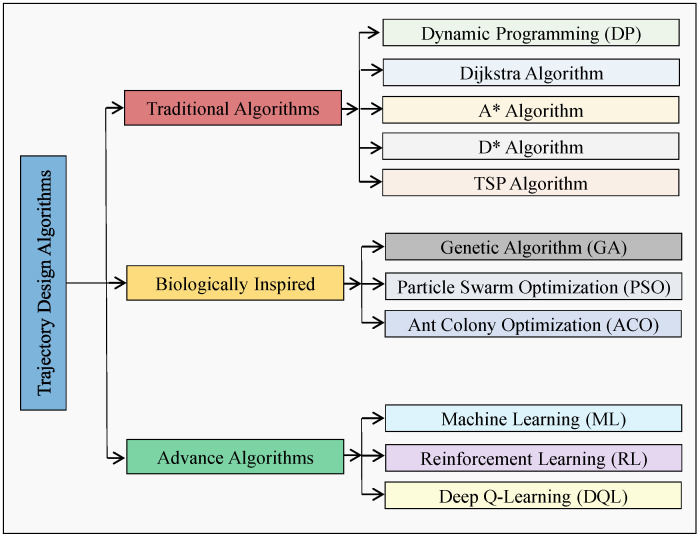
An overview of existing trajectory design algorithms.

Solving the trajectory design problem is a crucial and leading research topic in AI-enabled wireless UAV networks. This problem involves determining the optimal shortest path for a UAV to cover all targeted hotspot zones (nodes) in a dynamic wireless environment while adhering to time and mission completion constraints. This section discusses various techniques proposed in the literature for UAV trajectory design to optimize communication performance efficiently in a flexible wireless environment. These techniques can be categorized as classical and modern optimization algorithms as depicted in Figure 1.

In order to meet time constraints for all ground users, a feasible UAV trajectory was proposed in [43] using traditional dynamic programming (DP). However, due to an increase in hovering nodes, it may not align with time constraint criteria and may not be suitable for real-time environments. DP was also used to optimize the UAV trajectory in [44] for accessing multiple wireless sensor nodes (WSNs) and collecting data under time constraints. However, the algorithm was inefficient in recognizing and iterating through repeated grids, requiring high-order gridding for accuracy and resulting in computational complexity. In the study referenced as [45], the problem of the UAV trajectory was formulated as a mixed integer linear program (MILP). The trajectory planning is carried out in discrete time steps, where each step represents the dynamic state of the UAV in the environment. The algorithm is designed for offline planning to ensure a feasible trajectory is available before the UAV performs its tasks. However, this algorithm has limitations as it can easily become stuck due to its blind nature and cannot generate long trajectories in a complex environment. The Dijkstra algorithm proposed in [46] enables UAVs to perform environmental tasks efficiently by using the optimal battery level and reaching the target point in the shortest possible time. However, as the network scale increases, the algorithm takes a long time to provide a solution, making it unsuitable for real-time trajectory planning. The A* algorithm, as discussed in [47], selects suitable node pairs and evaluates the shortest path for UAVs based on feasible node pairs in a known static environment to address this issue. Although the A* algorithm does not provide a continuous path, it ensures that the shortest path is followed in the direction of the targeted node. However, this algorithm is not practical in a dynamic environment. To overcome this, the D* algorithm and its variants, as reviewed in [48], are efficient tools for quick re-planning in a cluttered environment. The D* algorithm updates the cost of new nodes, allowing the use of prior paths instead of re-planning the entire path. However, D* and its variants do not guarantee the quality of the solution in a large dynamic environment.

In order to design an effective path planning model for a UAV, the discrete space-based traveling salesman problem (TSP) [49] is utilized to search for the optimal shortest path for the UAV to travel through a fixed number of cities, with each city only being visited once. The UAV must also return to the starting city within a fixed flight time for battery charging. However, the TSP is an offline algorithm, so when a new city appears in the UAV’s path, the cost of the new city is updated from the starting point, resulting in the entire path being replanned from the start to the new end, which is a major drawback. The TSP is a challenging NP-hard problem and can be difficult to solve in polynomial time unless P = NP. Two approaches are available when dealing with the challenging NP-hard problem in TSP. The first involves using heuristics, such as 2-OPT and 3-OPT, to quickly generate near-optimal tours through local improvement algorithms [50]. The second approach is to utilize evolutionary optimization algorithms, such as genetic algorithm (GA), particle swarm optimization (PSO), and ant colony optimization (ACO), which have proven to be effective in minimizing the total distance travelled by the salesman in real-world scenarios [51]. While the GA is a good solution for obtaining an appropriate path for a UAV, it can be relatively slow, making it inefficient for modern path planning problems that require fast performance [52]. On the other hand, the PSO is good at local optimization and can be used in combination with a GA that is good at global optimization [53]. The ACO is also effective in solving the UAV path planning problem, but it requires a significant amount of data to find the optimal solution, has a slow iteration speed, and demands much more simulation time [54]. Therefore, a combination of these algorithms may be necessary to effectively solve the UAV path planning problem.

Reinforcement learning (RL) is a popular AI tool used to tackle complex problems such as trajectory design and sum-rate optimization, which are critical challenges due to the continuous environmental variation over time. Indeed, solving mathematical optimization models is only possible when a priori input data are available or requires too high complexity and computational time. Recent studies [55,56,57] proposed optimal trajectory design for UAVs using Q-learning to maximize the sum rate [55], increase the QoE of users [56], and enhance the number and fairness of users served [57]. However, Q-learning has a drawback in that the number of states increases exponentially with the number of input variables, and its memory usage also increases sharply. Due to the mobility of both ground and aerial users, the curse of dimensionality can cause Q-learning to fail. As a result, solving the trajectory design problem in a large and highly dynamic environment is a challenging task. A machine learning (ML) technique has been proposed in [58] to optimize the flight path of UAVs in order to meet the needs of ground users within specific zones during set time intervals. Another study in [59] explored a multi-agent Q-learning-based method to design the UAV’s flight path based on predicting the movement of the user to maximize the sum rate. Additionally, a meta-learning algorithm was introduced in [60] to optimize the UAV’s trajectory while meeting the uncertain and variable service demands of the GUs. However, these reinforcement learning-based solutions can only work in certain environments and are unsuitable for highly dynamic and unpredictable environments. A deep Q-learning (DQL) algorithm was introduced in [61] to enable UAVs to provide network service for ground users in rapidly changing environments autonomously. However, the user mobility model in this algorithm is simple and does not account for ground users moving to different positions multiple times, resulting in inadequate trajectory results for different paths.

In this work, we tackled the challenge of designing a UAV trajectory by treating it as a traveling salesman with profit problem (TSPWP). We leveraged the potent 2-OPT local search algorithm to attain an optimal offline solution. We then converted the resulting TSP instances from diverse examples into graphs and trained the UAV using them. This allowed the UAV to comprehend the properties of the TSP graphs and establish a world model that includes a hierarchical and multi-scale representation. This world model empowers the UAV to figure out the TSP strategy to solve the problem and implicitly discover the objective function. Our approach enables the UAV to deduce optimal routes by utilizing the beliefs encoded in the world model when confronted with a new realization. This significantly helps the UAV ascertain the best solution, even in situations where there are discrepancies between what it knows and what it sees.

## 3. System Model and Problem Formulation

**Figure 2:**
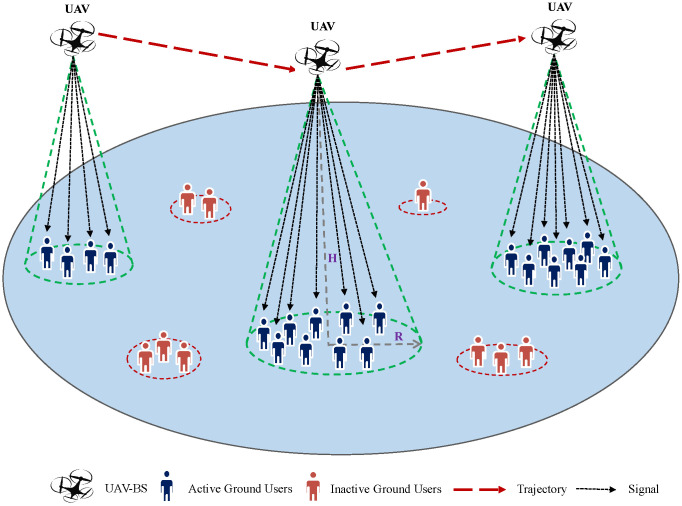
Illustration of the system model.

Consider a UAV-assisted wireless network, as shown in Figure 2, with a single UAV acting as a flying base station (FBS) to serve *U* ground users (GUs) distributed randomly across a geographical area and requesting uplink data service. GUs that demand the data service are introduced as active users; others are so-called inactive users, as illustrated in Figure 2. It is assumed that the GUs are partitioned into *N* distinct groups, each of which is defined as a hotspot area. The UAV’s mission is to fly from a start location, move towards hotspots with high data service requests, and then return to the initial location within a time period *T* for battery charging. Thus, the UAV’s initial ($l_{0}$) and final ($l_{T}$) locations are predefined, represented by $l_{0}=l_{T}=\left[x_{0},y_{0},z_{0}\right]$. It is important to note that the variable *T* is directly proportional to the number of available hotspots (*N*). As *N* increases, *T* also increases and vice versa. The UAV adjusts its deployment location at each flight slot according to the users realization forming a trajectory denoted by $q_{u}\left(t\right)=\left[x_{u}\left(t\right),y_{u}\left(t\right),z_{u}\left(t\right)\right]$. The sequence tracing UAV’s travels among the available hotspots during the flight time duration is given by ${\bar{q}}_{u}=\left[h_{1},\ldots ,h_{N^{\prime }}\right]$, where $h_{n}\in N$ is the *n*th hotspot served by the UAV and $N^{\prime }$ is the total number of the hotspots served along the trajectory. Let L be the set of all possible trajectories the UAV might follow and $\mathrm{Pr}(h_{n+1}|h_{n},\tau_{h_{n+1}})$ be the probability to move toward hotspot $h_{n+1}$ after being in $h_{n}$ (visited at time $T-\tau_{h_{n}}$), where $\tau_{h_{n+1}}$ is the remaining time to go back to the original location after serving $h_{n+1}$. The set of available hotspot areas is denoted as $N\overset{\Delta }{=}\left\{h_{n}=h_{1},h_{2},\ldots ,h_{N}\right\}$ and GUs across the total geographical area are denoted as $K\overset{\Delta }{=}\left\{K_{n}=K_{1},K_{2},\ldots ,K_{N}\right\}$, where $K_{n}$ is the set of users belonging to the *n*th hotspot and each GU belongs to a single hotspot where the coordinate of each GU is given by $p_{k_{n}}=\left[x_{k_{n}},y_{k_{n}}\right]$. Each hotspot *n* is characterized by its center $p_{n}=\left[x_{n},y_{n}\right]$ and radius $r_{n}$ representing the coverage range and the average data rate $R_{n}$ that depends on the number of active users in hotspot *n* where $R_{n}\in R$, such that $R\overset{\Delta }{=}\left\{R_{n}=R_{1},R_{2},\ldots ,R_{N}\right\}$.

To capture the dynamic nature of the network, the UAV flight time (*T*) is discretized into a set T of *M* equal time slots where the length of each time slot is $t=(\frac{T}{M})$. Due to its short duration, the UAV’s location, uplink data requests and channel conditions are considered fixed in each *t*. Furthermore, in the considered network, the UAV assigns a set of uplink resource blocks (RBs) to serve the active GUs in a specific hotspot (one RB for each active GU) who transmit their data over the allocated RBs using the orthogonal frequency division multiple access (OFDMA) scheme.

In our network, the air-to-ground signal propagation is adopted and a probabilistic path loss model subject to random line-of-sight (LoS) and non-line-of-sight (NLOS) conditions is considered [62]. The channel gain between a GU ($k_{n}\in K_{n}$) and a UAV (*u*) can be expressed as:

(1) $$g_{k_{n},u}\left(t\right)=\frac{1}{K_{0}d_{k_{n},u}^{\alpha }\left(t\right)}{\left[{\mathrm{Pr}}_{\mathrm{LoS}}\mu_{\mathrm{LoS}}+{\mathrm{Pr}}_{\mathrm{NLoS}}\mu_{\mathrm{NLoS}}\right]}^{-1},$$

where $K_{0}={\left(\frac{4\pi f_{c}}{c}\right)}^{2}$, $f_{c}$ is the carrier frequency, *c* is the speed of light, α is the path loss exponent, and ${\mathrm{Pr}}_{\mathrm{LoS}}$ and ${\mathrm{Pr}}_{\mathrm{NLoS}}$ are the LoS and NLoS probabilities, respectively. $\mu_{\mathrm{LoS}}$ and $\mu_{\mathrm{NLoS}}$ are additional attenuation factors to the free-space propagation for LoS and NLoS links, respectively. The distance between a GU ($k_{n}$) and the UAV at time slot *t* is given by:

(2) $$d_{k_{n},u}\left(t\right)=\sqrt{h_{u}{\left(t\right)}^{2}+{\left(x_{k_{n}}\left(t\right)-x_{u}\left(t\right)\right)}^{2}+{\left(y_{k_{n}}\left(t\right)-y_{u}\left(t\right)\right)}^{2}}.$$

The average achievable data rate of the set of users in hotspot *n* is calculated as:

(3) $$r_{K_{n}}=\sum_{k_{n}=1}^{K_{n}}r_{k_{n}}=\sum_{k_{n}=1}^{K_{n}}B_{k_{n}}\log_{2}\left(1+\frac{p_{k_{n}}g_{k_{n},u}\left(t\right)}{\sigma^{2}}\right),$$

where $B_{k_{n}}$ is the bandwidth of the RB allocated to GU ($k_{n}$), $p_{k_{n}}$ is the transmit power of GU ($k_{n}$), and $\sigma^{2}=B_{k_{n}}N_{0}$ is the power spectral density of the additive white Gaussian noise (AWGN).

In this work, we focus on UAV trajectory design that can maximize the total sum-rate in the cell. Therefore, our optimization objective can be formulated as:

(4a) $$\begin{array}{cc} \max_{q_{u}\in L}\;r_{sum} & =\sum_{h_{n}=1}^{N^{\prime }}\sum_{k_{n}=1}^{K_{n}}r_{k_{n}}\prod_{h_{n}=1}^{N^{\prime }-1}\mathrm{Pr}\left(h_{n+1}|h_{n},\tau_{h_{n+1}}\right) \end{array}$$

(4b) $$\begin{array}{cc} s.t. & k_{i}\cap k_{j}=\phi ,\;i\neq j,\;\forall i,j\in N, \end{array}$$

(4c) $$\begin{array}{cc} \; & \;t\left(q_{u}\right)\leq T,\;q_{u}\in L, \end{array}$$

(4d) $$\begin{array}{cc} \; & \;0\leq \mathrm{Pr}(h_{n+1}|h_{n},\tau_{h_{n+1}})\leq 1,\;1\leq h_{n}\leq N^{\prime }-1, \end{array}$$

(4e) $$\begin{array}{cc} \; & \;r_{k_{n}}\geq r_{0},\;\forall k_{n}, \end{array}$$

(4f) $$\begin{array}{cc} & 0\leq p_{k_{n}}\leq p_{max},\;\forall k_{n}. \end{array}$$

Constraint (4b) indicates that each GU belongs to a specific hotspot. (4c) implies that the UAV must go back to the initial location before *T*, where *T* is directly proportional to *N*. If *N* increases, *T* will also increase; if *N* decreases, *T* will also decrease. Furthermore, (4e) represents the sum-rate requirement for each GU and (4f) depicts the power allocation constraint. It is worth noting that in this paper, the number of hotspots remains constant in a certain mission (realization). No new hotspots emerge nor do any existing hotspots disappear while the UAV is solving a specific realization.

The symbols used in the article and their meanings are summarized in Table 1.

## 4. Proposed Goal-Directed Trajectory Design Method

In this section, we propose a goal-directed method for UAV trajectory design based on active inference. The latter is a model-based data-driven approach that rests upon the idea of using an internal generative model (world model) to cast the surrounding environment and planning actions allowing realization goals to be targeted by the agent. First, we present the perceptual learning of desired observation based on a classical traveling salesman problem (TSP) with 2-OPT [63]. Then, we show how to build the world model representing the surrounding environment by encoding the dynamic rules behind the optimal TSP trajectories.

### 4.1. TSP with Profits Instances

The traditional TSP is a classic algorithm problem in computer science and operation research describing how a salesman travels to several vertices (cities) and returns to the terminal (initial location), aiming to minimize the travel cost (i.e., the travel distance) while ensuring visiting each city only once [63]. In this work, we adopt the TSP with profits (TSPWP) with the 2-OPT local search algorithm [39], which is a generalization of the traditional TSP where the overall goal is the simultaneous optimization of the collected profit and the travel cost, knowing that each vertex (city) is associated with a profit. Thus, TSPWP is used to generate optimal trajectory instances *offline* that the UAV might follow to serve more users within a predefined time. Given a list of hotspots where the active users are distributed, as shown in Figure 2, and the cost ($c_{ij}$) of transiting between each pair of hotspots, the problem is to find the optimal route that visits each hotspot once and returns to the origin, providing a maximum sum-rate and a minimum completion time.

Let G=(V,E) be a graph where $V=\{v_{1},\ldots ,v_{N}\}$ is a set of *N* vertices and E is a set of edges. Let $p_{n}$ be the center of $v_{n}$ and $r_{K_{n}}$ the profit associated with $v_{n}$ and a cost $c_{ij}$ be associated with each edge $(v_{i},v_{j})\in E$, such that:

(5) $$c_{ij}=d\left(p_{i},p_{j}\right)=\sqrt{{\left(x_{i}-x_{j}\right)}^{2}+{\left(y_{i}-y_{j}\right)}^{2}}.$$

The objective function of the TSPWP with *N* hotspots can be defined as:

(6a) $$\begin{array}{cc} \min\; & \alpha \sum_{(v_{i},v_{j})\in E}c_{ij}x_{ij}-\beta \sum_{v_{j}\in V}r_{K_{j}}y_{j}, \end{array}$$

(6b) $$\begin{array}{cc} s.t.\; & \sum_{\begin{array}{c} v_{i}\in V\\ v_{j}\in V\setminus \left\{v_{i}\right\} \end{array}}x_{ij}=y_{i}, \end{array}$$

(6c) $$\begin{array}{cc} \; & \sum_{\begin{array}{c} v_{j}\in V\\ v_{i}\in V\setminus \left\{v_{j}\right\} \end{array}}x_{ij}=y_{j}, \end{array}$$

(6d) $$\begin{array}{cc} \; & x_{ij}\in \left\{0,1\right\},\;\left(v_{i},v_{j}\right)\in E, \end{array}$$

(6e) $$\begin{array}{cc} \; & y_{ij}\in \left\{0,1\right\},\;\left(v_{i}\in V\right), \end{array}$$

(6f) $$\begin{array}{cc} \; & \alpha +\beta =1. \end{array}$$

Constraints (6b) and (6c) are the assignment constraints where $x_{ij}$ is a binary variable associated with edge ($v_{i}$, $v_{j}$), equal to 1 if and only if ($v_{i}$, $v_{j}$) is used in the solution, and $y_{i}$ is a binary variable associated with vertex $v_{i}\in V$, equal to 1 if and only if $v_{i}$ is visited.

### 4.2. World Model

The proposed approach consists of two computational units. The first unit aims to learn the surrounding environment by representing the statistical structure of the sensory signals (world model). The second is the decision-making unit seeking to perform actions minimizing (or maximizing) a cost function describing preferred outcomes (similar to rewards in RL). The world model is an internal generative model representing the surrounding environment (both physical and wireless environment) utilized by the UAV to make predictions about incoming sensory signals. In this subsection, given the TSPWP instances generated previously from several experiences (i.e., realizations of users distribution and users requests), our objective is to encode the dynamic rules generating those instances in a probabilistic graphical model capable of reflecting the graph structure of the TSPWP instances at multiple hierarchical levels and different time scales.

#### 4.2.1. Dictionary Learning

**Figure 3:**
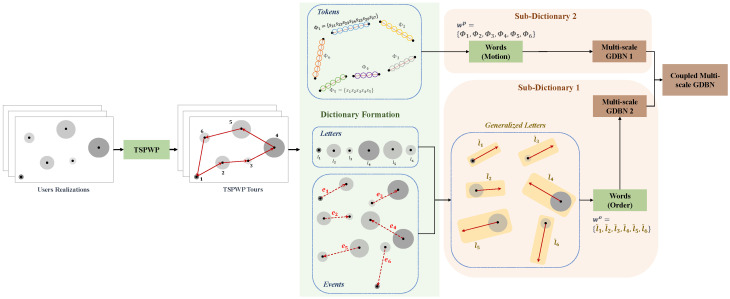
The procedure to form the global dictionary.

Each TSPWP instance comprises the trajectory the UAV follows to reach the targeted hotspots in a particular order. Hence, the objective is to form a dictionary capturing the TSPWP graph structure, allowing one to predict the most probable hotspot to target conditioned on a specific location and the most probable path to follow to reach that targeted hotspot. Thus, the dictionary consists of two sub-dictionaries. The first encodes the rules generated the sequence order of the hotspots that the UAV intends to serve. By contrast, the second sub-dictionary encodes the rules generated the motion to travel among to neighboring hotspots. Figure 3 illustrates the process of forming the global dictionary.

**(1) TSPWP offline execution:**

Let $D\overset{\Delta }{=}\left\{D_{m}=D_{1},D_{2},\ldots ,D_{M}\right\}$ be a training set of realizations representing *M* examples of users’ distribution in the cell, where $D_{m}$ is the *m*-th realization and *M* is the total number of realizations. Each realization consists of the number of hotspots and their locations, the number of users inside each hotspot as well as the users’ access request and users’ locations. The TSPWP algorithm will be employed offline to solve all the examples in D. Consequently, let $L^{†}\overset{\Delta }{=}\left\{L_{m}=L_{1},L_{2},\ldots ,L_{M}\right\}$ be a set of the sequences of hotspots selected by the UAV using TSPWP to solve the *M* examples, where $L_{m}=\left\{h_{1},\ldots ,h_{N^{\prime }}\right\}$ is the *m*-th sequence of hotspots selected by the UAV to solve the *m*-th example and let $Q^{†}\overset{\Delta }{=}\left\{q_{u}^{m}=q_{u}^{1},q_{u}^{2},\ldots ,q_{u}^{M}\right\}$ be the set of trajectory instances generated by the TSPWP, where $q_{u}^{m}$ is the *m*-th TSPWP trajectory generated to solve the *m*-th example.

**(2) Unsupervised Clustering:**

For each of the generated trajectories in $Q^{†}$, a growing neural gas (GNG) is employed on the generalized errors (GEs) provided by the unmotivated Kalman filter (UKF) [64] to discover the dynamic rules driving the different trajectories. Let S be the set of clusters generated by GNG and defined as:

(7) $$S\overset{\Delta }{=}\left\{s_{f}=s_{1},s_{2},\ldots ,s_{F}\right\},$$

where $s_{f}$ is the *f*-th cluster following a Gaussian distribution such that $s_{f}∼N\left(\mu_{s_{f}},\Sigma_{s_{f}}\right)$, and *F* is the total number of clusters. Clustering the trajectory data allows obtaining knowledge that reveals the latent characteristics of the UAV’s motion.

**(3) Sub-Dictionary 1:**

Accordingly, from $L^{†}$ we form a sub-dictionary encoding the decisions made by the UAV consisting of the sequences of targeted hotspots. We define a letter $l_{m}=h_{m}$ representing a starting hotspot $h_{m}$ at a given time and a generalized letter defined as:

(8) $${\tilde{l}}_{m}=\left[h_{m},E\left(h_{m},h_{m^{\prime }}\right)\right],$$

consisting of the letter itself and its derivative illustrating the event of traveling from hotspot $h_{m}$ to hotspot $h_{m^{\prime }}$. It is of note that a generalized letter ${\tilde{l}}_{m}$ can be seen as a pair of one node $n_{i}=h_{m}$ and one outgoing arc $\left(n_{i},n_{j}\right)$ from node $n_{i}$ to node $n_{j}$, as shown in Figure 3. Then, for each element $L_{m}$ in $L^{†}$, we transform the sequence of generalized letters expressing that experience into the following sequence: $\left\{{\tilde{l}}_{m,\tau_{1}},{\tilde{l}}_{m,\tau_{2}},\ldots ,{\tilde{l}}_{m,\tau_{T}}\right\}$ describing the transitions between adjacent event-steps. As mentioned before, the generalized letters of a certain experience *m* can be seen as an unweighted graph $G_{m}=\left(V_{m},E_{m}\right)$, where $V_{m}=\left\{l_{m,\tau_{1}},\ldots ,l_{m,\tau_{T}}\right\}$ is a set of vertices represented by the letters and $E_{m}=\left\{{\dot{l}}_{m,\tau_{1}},\ldots ,{\dot{l}}_{m,\tau_{T}}\right\}$ is the set of edges represented by the letters’ derivatives. The adjacency matrix $A_{{\tilde{l}}_{m}}$ that captures the pattern of co-occurrences in the generalized letters sequence is an $\tau_{T}\times \tau_{T}$ zero–one matrix defined as $A_{{\tilde{l}}_{m}}=\left[a_{ij}\right]$, where:

(9) $$a_{ij}=\left\{\begin{array}{c} 1\;\mathrm{if}\;(i,j)\in E,\\ 0\;\mathrm{Otherwise}. \end{array}\right.$$

After executing the *M* examples, we can form the global adjacency matrix $A_{\tilde{l}}=\left[a_{i^{\prime },j^{\prime }}\right]$ comprising all the generalized letters (forming a global graph $G_{global}=\left(V_{global},E_{global}\right)$) that occurred while solving the *M* examples, such that:

(10) $$a_{i^{\prime },j^{\prime }}=\left\{\begin{array}{c} 1\;\mathrm{if}\;\left(i^{\prime },j^{\prime }\right)\in E_{global},\\ 0\;\mathrm{Otherwise}. \end{array}\right.$$

Where element $a_{i^{\prime },j^{\prime }}$ denotes the number of times that a generalized letter ${\tilde{l}}_{i^{\prime }}$ is followed by generalized letter ${\tilde{l}}_{j^{\prime }}$ during two consecutive events in the global graph $G_{global}$.

The degree of each letter $i=l_{m,\tau_{i}}$ is the number of its adjacent letters (or the number of outgoing edges at that letter) calculated as: $d_{i}=\sum_{j=1}^{|V_{m}|}a_{i^{\prime }j^{\prime }}$. Considering the degrees of all letters, we can construct the degree matrix D, which is an $|V_{m}\left|\times \right|V_{m}|$ diagonal matrix defined as:

(11) $$D_{i^{\prime }j^{\prime }}=\left\{\begin{array}{c} d_{i^{\prime }}\;\mathrm{if}\;i^{\prime }=j^{\prime },\\ 0\;\mathrm{Otherwise}. \end{array}\right.$$

Consequently, the global transition matrix can be constructed in the following way:

(12) $$\Pi_{\tilde{l}}=D^{-1}A_{\tilde{l}}=\left[\begin{array}{cccc} \mathrm{Pr}({\tilde{l}}_{1}|{\tilde{l}}_{1}) & \mathrm{Pr}({\tilde{l}}_{1}|{\tilde{l}}_{2}) & \ldots & \mathrm{Pr}({\tilde{l}}_{1}|{\tilde{l}}_{M^{\prime }})\\ \mathrm{Pr}({\tilde{l}}_{2}|{\tilde{l}}_{1}) & \mathrm{Pr}({\tilde{l}}_{2}|{\tilde{l}}_{2}) & \ldots & \mathrm{Pr}({\tilde{l}}_{2}|{\tilde{l}}_{M^{\prime }})\\ \vdots & \vdots & \vdots & \vdots \\ \mathrm{Pr}({\tilde{l}}_{M^{\prime }}|{\tilde{l}}_{1}) & \mathrm{Pr}({\tilde{l}}_{M^{\prime }}|{\tilde{l}}_{2}) & \ldots & \mathrm{Pr}({\tilde{l}}_{M^{\prime }}|{\tilde{l}}_{M^{\prime }}) \end{array}\right],$$

where $0\leq \mathrm{Pr}({\tilde{l}}_{\tilde{i}}|{\tilde{l}}_{\tilde{j}})\leq 1$ and $\sum_{\tilde{j}=1}^{\tilde{J}}\mathrm{Pr}\left({\tilde{l}}_{\tilde{i}}|{\tilde{l}}_{\tilde{j}}\right)=1,\forall \tilde{j}$. During a flight mission that lasts for a time period *T*, the order of visited hotspots is recorded in a word called $w_{T}^{o}=\left\{{\tilde{l}}_{m,\tau_{1}},{\tilde{l}}_{m,\tau_{2}},\ldots ,{\tilde{l}}_{m,\tau_{T}}\right\}$.

**(4) Sub-Dictionary 2:**

Each event $e_{m}=E\left(h_{m},h_{m^{\prime }}\right)$ can be associated with a local trajectory followed by the UAV to pass from $h_{m}$ to $h_{m^{\prime }}$, which can be represented by a sequence of discrete clusters. This is possible after associating the local trajectory with S defined in (7) to form a token comprising a sequence of letters depicting the firing sequence of clusters (neurons) from S during a certain event, i.e, $e_{m}$. Hence, we define a token consisting of a set of clusters and representing a local path between two adjacent hotspots as follows:

(13) $$\Theta_{e_{m}}=\left\{s_{e_{m},t_{1}},s_{e_{m},t_{2}},\ldots ,s_{e_{m},t_{\tau }}\right\},$$

where $s_{e_{m},t_{i}}\in S$, and $t_{\tau }$ is the duration of event $e_{m}$ specified in the number of time slots. The stochastic process decomposing the interdependent nature of the tokens that make up the local trajectories can be illustrated in a transition matrix defined as:

(14) $$\Pi_{\Theta }=\left[\begin{array}{cccc} \mathrm{Pr}(\Theta_{e_{1}}|\Theta_{e_{1}}) & \mathrm{Pr}(\Theta_{e_{1}}|\Theta_{e_{2}}) & \ldots & \mathrm{Pr}(\Theta_{e_{1}}|\Theta_{e_{M}})\\ \mathrm{Pr}(\Theta_{e_{2}}|\Theta_{e_{1}}) & \mathrm{Pr}(\Theta_{e_{2}}|\Theta_{e_{2}}) & \ldots & \mathrm{Pr}(\Theta_{e_{2}}|\Theta_{e_{M}})\\ \vdots & \vdots & \vdots & \vdots \\ \mathrm{Pr}(\Theta_{e_{M}}|\Theta_{e_{1}}) & \mathrm{Pr}(\Theta_{e_{M}}|\Theta_{e_{2}}) & \ldots & \mathrm{Pr}(\Theta_{e_{M}}|\Theta_{e_{M}}) \end{array}\right],$$

where $\mathrm{Pr}(\Theta_{e_{i}}|\Theta_{e_{j}})$ depicts the transition probability from token *i* to token *j*, such that $0\leq \mathrm{Pr}(\Theta_{e_{i}}|\Theta_{e_{j}})\leq 1$ and $\sum_{j=1}^{J}\mathrm{Pr}\left(\Theta_{e_{i}}|\Theta_{e_{j}}\right)=1,\forall j$. During a flight mission of duration *T*, the tokens that represent the entire trajectory are recorded in a word called $w_{T}^{p}=\left\{\Theta_{e_{j}},\Theta_{e_{j+1}},\ldots ,\Theta_{e_{J}}\right\}$.

#### 4.2.2. The Proposed Hierarchical Graphical Representation

**Introducing Multi-Scale GDBN:** We can see that the UAV’s dynamic behavior manifests at multiple time scales, namely slot scale and event scale. It is essential to have an efficient representation that can model this dynamic behavior, including a hierarchical structure and incorporating Markov chains at various time scales. To achieve this, we propose to learn two separated dynamic models representing the dynamic behavior of the UAV when selecting the targeted hotspots (i.e., the sequence of hotspots to serve during the flight time) and when moving between two consecutive hotspots (i.e., the UAV’s motion path). The proposed representation considers observations stemming from two different behavioral processes with different temporal resolutions. The first process determines the decisions made by the UAV at the event scale, while the second process determines the UAV’s motion at the finer time scale (slot scale), which is nested within the event scale.

**Figure 4:**
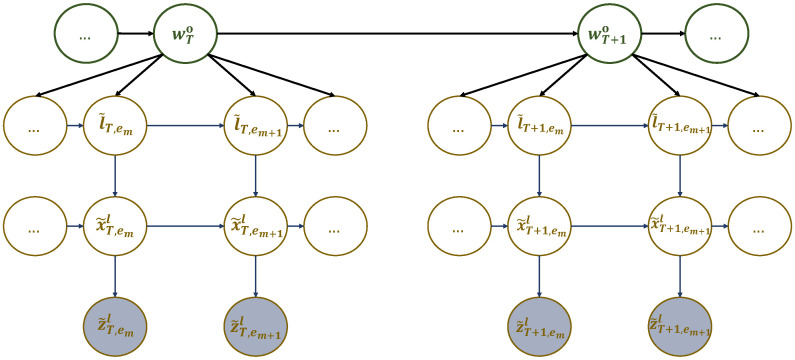
A multi-scale GDBN representing sub-dictionary 1 that encodes the dynamic rules generating UAV’s hotspots sequence in different experiences.

The first dynamic model entails arranging particular elements of the dictionary (sub-dictionary 1), particularly the generalized letters referenced in (8), into a multi-scale generalized dynamic Bayesian network (M-GDBN) displayed in Figure 4. The M-GDBN is a hierarchical probabilistic graphical model that consists of four levels, two of which are continuous and two of which are discrete. Each level corresponds to a distinct hierarchy and time scale. Furthermore, M-GDBN explains how the latent state variables and the observation are probabilistically linked. The explanation for the evolution of hidden variables at multiple levels is provided based on the following dynamic models:

(15a) $$\begin{array}{cc} \; & w_{T}^{o}=f^{\left(1\right)}\left(w_{T-1}^{o}\right)+\eta_{T}, \end{array}$$

(15b) $$\begin{array}{cc} \; & {\tilde{l}}_{T,e_{m}}=f^{\left(2\right)}\left({\tilde{l}}_{T,e_{m-1}},w_{T}^{o}\right)+\eta_{T,e_{m}}, \end{array}$$

(15c) $$\begin{array}{cc} \; & {\tilde{x}}_{T,e_{m}}^{l}=g^{\left(1\right)}\left({\tilde{x}}_{T,e_{m-1}}^{l},{\tilde{l}}_{T,e_{m}}\right)+\eta_{T,e_{m}}, \end{array}$$

(15d) $$\begin{array}{cc} \; & {\tilde{z}}_{T,e_{m}}^{l}=g^{\left(2\right)}\left({\tilde{x}}_{T,e_{m}}^{l}\right)+\nu_{T,e_{m}}. \end{array}$$

The discrete state equations in (15a) and (15b) illustrate how words and generalized letters change over time at various temporal scales. $f^{\left(1\right)}$ and $f^{\left(2\right)}$ are nonlinear functions that experience random fluctuations in the states influenced by higher levels and characterized by $\eta_{T}∼N\left(0,Q\right)$ and $\eta_{T,e_{m}}∼N\left(0,Q\right)$. Going down the hierarchy, Equations (15c) and (15d) stand for the continuous state equation and the observation model, explaining the continuous state dynamic evolution and the mapping from the continuous state space to the measurement space, respectively. Observations are subject to random fluctuations playing the role of observation noise characterized by $\nu_{T,e_{m}}∼N\left(0,\sigma_{{\tilde{z}}_{T,e_{m}}}^{2}\right)$. Equations (15a), (15b), (15c), and (15d) can be expressed in probabilistic form as $\mathrm{Pr}(w_{T}^{o}|w_{T-1}^{o})$, $\mathrm{Pr}({\tilde{l}}_{T,e_{m}}|{\tilde{l}}_{T,e_{m-1}},w_{T}^{o})$, $\mathrm{Pr}({\tilde{x}}_{T,e_{m}}^{l}|{\tilde{x}}_{T,e_{m-1}}^{l},{\tilde{l}}_{T,e_{m}})$, and $\mathrm{Pr}({\tilde{z}}_{T,e_{m}}^{l}|{\tilde{x}}_{T,e_{m-1}}^{l})$, respectively. Thus, the consistent global model (i.e., the joint distribution function) corresponding to the network in Figure 4 is given by:

(16) $$\mathrm{Pr}\left(W^{o},\tilde{L},{\tilde{X}}^{l},{\tilde{Z}}^{l}\right)=\prod_{T}\mathrm{Pr}\left(w_{T}^{o}\right)\prod_{T,e_{m}}\mathrm{Pr}\left({\tilde{l}}_{T,e_{m}}|w_{T}^{o}\right)\mathrm{Pr}\left({\tilde{x}}_{T,e_{m}}^{l}|{\tilde{l}}_{T,e_{m}}\right)\mathrm{Pr}\left({\tilde{z}}_{T,e_{m}}^{l}|{\tilde{x}}_{T,e_{m}}^{l}\right).$$

M-GDBN is a directed acyclic graph where every node represents a random variable or uncertain quantity that can have multiple values. The arcs indicate a direct causal influence between linked variables, and the strength of these influences is measured by conditional probabilities. To determine the structure of M-GDBN, a node is assigned to each variable, and arrows are drawn towards it from nodes that are perceived to be its direct cause. To determine the strength of direct influences, each variable is assigned a link matrix. This matrix represents the estimated conditional probabilities of the event based on the parent set’s value combination.

**Figure 5:**
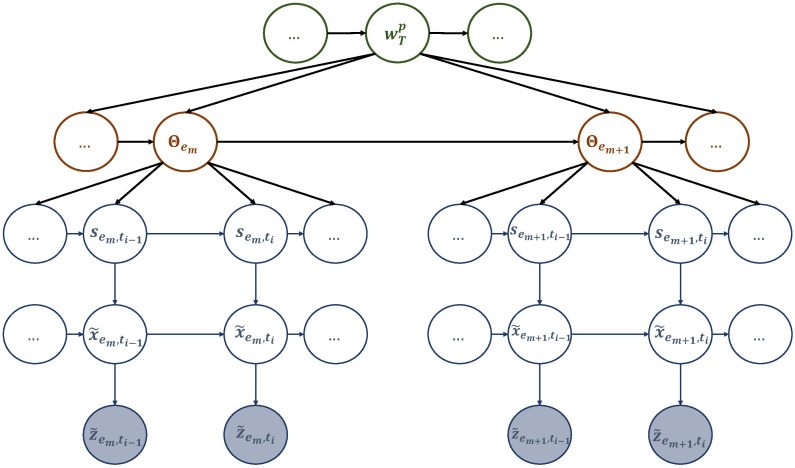
A multi-scale GDBN representing sub-dictionary 2 that encodes the dynamic rules generating the UAV’s positions to travel among the hotspots in different events.

In Figure 5, there is another multi-scale GDBN that deals with the dictionary components concerning the UAV’s dynamic motion (sub-dictionary 2). This second network has three discrete levels and two continuous levels. The variables at the various levels explain how the observations (i.e., the UAV’s trajectory) were generated. For instance, at the word scale, each word is made up of tokens that were realized at different events (event scale). Each token, in turn, is composed of discrete and continuous letters that generate observations at different slots.

In order to comprehend the generative process forming the UAV’s global trajectory, we can refer to the dynamic models below:

(17a) $$\begin{array}{cc} \; & w_{T}^{p}=f^{\left(1\right)}\left(w_{T-1}^{p}\right)+\eta_{T}, \end{array}$$

(17b) $$\begin{array}{cc} \; & \Theta_{T,e_{m}}=f^{\left(2\right)}\left(\Theta_{T,e_{m-1}},w_{T}^{p}\right)+\eta_{T}, \end{array}$$

(17c) $$\begin{array}{cc} \; & {\tilde{s}}_{e_{m},t_{i}}=f^{\left(3\right)}\left({\tilde{s}}_{e_{m},t_{i-1}},\Theta_{T,e_{m}}\right)+\eta_{T,e_{m}}, \end{array}$$

(17d) $$\begin{array}{cc} \; & {\tilde{x}}_{e_{m},t_{i}}=g^{\left(1\right)}\left({\tilde{x}}_{e_{m-1},t_{i-1}},{\tilde{s}}_{e_{m},t_{i}}\right)+\eta_{e_{m},t_{i}}, \end{array}$$

(17e) $$\begin{array}{cc} \; & {\tilde{z}}_{e_{m},t_{i}}=g^{\left(2\right)}\left({\tilde{x}}_{e_{m},t_{i}}\right)+\nu_{e_{m},t_{i}}. \end{array}$$

The discrete state equations in (17a), (17b), and (17c) show how the trajectory words, tokens, and trajectory clusters change over time at various temporal scales. These equations use non-linear functions $f^{\left(1\right)}$, $f^{\left(2\right)}$, and $f^{\left(3\right)}$ subject to process noise $\eta_{T}∼N\left(0,Q\right)$. The continuous state equation in (17d) explains how the trajectory states evolve over time, while (17e) links observations to these states. The equations mentioned earlier can be expressed probabilistically as follows: $\mathrm{Pr}(w_{T}^{p}|w_{T-1}^{p})$, $\mathrm{Pr}(\Theta_{T,e_{m}}|\Theta_{T,e_{m-1}},w_{T}^{p})$, $\mathrm{Pr}({\tilde{s}}_{e_{m},t_{i}}|{\tilde{s}}_{e_{m},t_{i-1}},\Theta_{T,e_{m-1}})$, $\mathrm{Pr}({\tilde{x}}_{e_{m},t_{i}}|{\tilde{x}}_{e_{m},t_{i-1}},{\tilde{s}}_{e_{m},t_{i}})$, and $\mathrm{Pr}({\tilde{z}}_{e_{m},t_{i}}|{\tilde{x}}_{e_{m},t_{i}})$, respectively. The network in Figure 5 has a compatible global model, represented by a joint distribution function that can be expressed as:

(18) $$\mathrm{Pr}\left(W^{p},\Phi ,\tilde{S},\tilde{X},\tilde{Z}\right)=\prod_{T}\mathrm{Pr}\left(w_{T}^{p}\right)\prod_{T,e_{m}}\mathrm{Pr}\left(\Theta_{T,e_{m}}|w_{T}^{p}\right)\mathrm{Pr}\left({\tilde{s}}_{T,e_{m}}|{\tilde{x}}_{T,e_{m}}\right)\mathrm{Pr}\left({\tilde{z}}_{T,e_{m}}|{\tilde{x}}_{T,e_{m}}\right).$$

**Coupled-MGDBN:** We have organized the dictionaries we obtained into a coupled multi-scale generalized dynamic Bayesian network (C-MGDBN), which includes the two dynamic models. The first model represents the sequence of hotspots the UAV selects to solve the realizations encountered during training, which is structured in sub-dictionary 1. Meanwhile, the second model represents the UAV’s path to travel between consecutive hotspots, which is structured in sub-dictionary 2. By coupling these two models stochastically in the C-MGDBN, we can incorporate more complex and sophisticated dynamics and model stochastic representations of multiple behaviors. Additionally, we have added an efficient mechanism to the C-MGDBN that captures multiple event and state transitions, which help explain how the UAV approached a particular task (such as trajectory design) in different examples.

**Figure 6:**
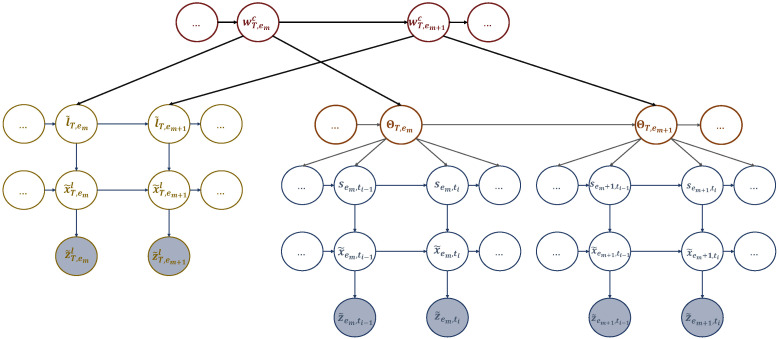
A coupled multi-scale GDBN (C-MGDBN) structures the acquired dictionaries by coupling the corresponding models at the event scale.

We coupled the two M-GDBN models mentioned earlier at the event scale as shown in Figure 6. This was done because multiple events make up a complete mission. We have yet to investigate coupling at the word scale. However, this coupling technique can be useful if the UAV is performing various missions. For instance, after serving active users in a specific cell, the UAV can return to its initial station for recharging before proceeding to another mission. In this way, by learning the dynamics of real-life scenarios, which include users’ activities and the emergence of hotspots, the UAV can plan its actions at the word scale. For the rest of the paper, we will assume that the UAV is making plans at both the event and slot scales.

In the C-MGDBN depicted in Figure 6, the current discrete state is influenced by the state of its own chain and that of the neighboring chain from the previous event step. To avoid overwhelming complexity, we conducted a meta-clustering process by merging dependent nodes in the connected network into a single higher-dimensional node. In other words, $\mathrm{Pr}(\Theta_{T,e_{m+1}}|\Theta_{T,e_{m}},{\tilde{l}}_{T,e_{m}})$, and vice versa $\mathrm{Pr}({\tilde{l}}_{T,e_{m+1}}|{\tilde{l}}_{T,e_{m}},\Theta_{T,e_{m}})$. To estimate these probabilities we need two transition matrices encoding the probabilistic relationships between words and tokens. Merging letters and tokens allows to simplify the case by coupling them into a higher node $w_{T,e_{m}}^{c}=\left[{\tilde{l}}_{T,e_{m}},\Theta_{T,e_{m}}\right]$. The evolution of the coupling words $w_{T,e_{m}}^{c}$ can be captured by the transition matrix defined as:

(19) $$\Pi_{w^{c}}=\left[\begin{array}{cccc} \mathrm{Pr}(w_{1}^{c}|w_{1}^{c}) & \mathrm{Pr}(w_{1}^{c}|w_{2}^{c}) & \ldots & \mathrm{Pr}(w_{1}^{c}|w_{C}^{c})\\ \mathrm{Pr}(w_{2}^{c}|w_{1}^{c}) & \mathrm{Pr}(w_{2}^{c}|w_{2}^{c}) & \ldots & \mathrm{Pr}(w_{2}^{c}|w_{C}^{c})\\ \vdots & \vdots & \vdots & \vdots \\ \mathrm{Pr}(w_{C}^{c}|w_{1}^{c}) & \mathrm{Pr}(w_{C}^{c}|w_{2}^{c}) & \ldots & \mathrm{Pr}(w_{C}^{c}|w_{C}^{c}) \end{array}\right],$$

where $0\leq \mathrm{Pr}(w_{i}^{c}|w_{j}^{c})\leq 1$ and $\sum_{j=1}^{J}\mathrm{Pr}\left(w_{i}^{l}|w_{j}^{c}\right)=1,\forall j$. $\Pi_{w^{c}}$ can be considered as a combined transition matrix, formed by coupling (12) with (14).

### 4.3. Active Inference

**Figure 7:**
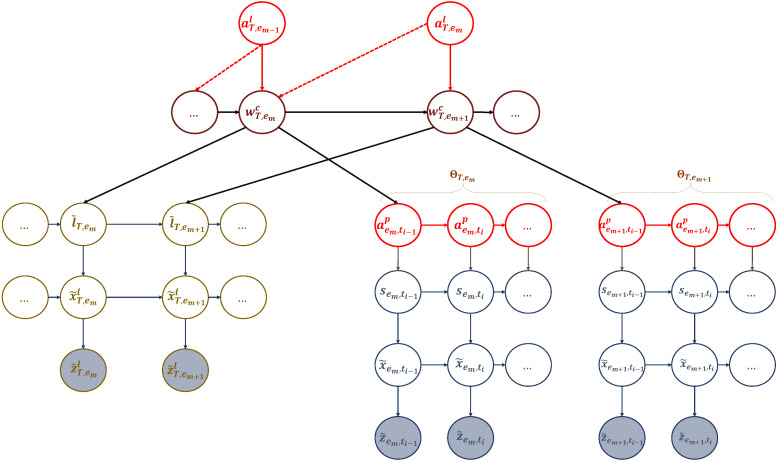
An active multi-scale GDBN involving the active states representing the actions that the UAV can perform and affect the dynamic rules generating UAV’s positions to travel among the hotspots in different events.

During the active inference process, a UAV can learn, adapt, and perceive its body as a unit while interacting with the environment. The UAV’s world model can be defined as a partially observable Markov decision process (POMDP). It involves a probability distribution $\mathrm{Pr}(Z^{l},Z,{\tilde{X}}^{l},\tilde{X},S,\tilde{L},A^{l},A^{p},W)$ that determines the joint probability of the UAV’s observations, belief states, actions, and words (i.e., policies). In simpler terms, a word (or policy) refers to a set of actions. This concept is illustrated through events in Figure 7, and it can be expressed in the following format:

(20) $$\begin{array}{c} \mathrm{Pr}\left(Z^{l},Z,{\tilde{X}}^{l},\tilde{X},S,\tilde{L},A^{l},A^{p},W\right)=\mathrm{Pr}\left({\tilde{l}}_{0}\right)\mathrm{Pr}\left({\tilde{x}}_{0}^{l}\right)\mathrm{Pr}\left(w_{0}^{c}\right)\\ \prod_{e_{m}=1}^{E_{m}}\mathrm{Pr}\left({\tilde{z}}_{e_{m}}^{l}|{\tilde{x}}_{e_{m}^{l}}\right)\mathrm{Pr}\left({\tilde{x}}_{e_{m}}^{l}|{\tilde{l}}_{e_{m}}\right)\mathrm{Pr}\left({\tilde{l}}_{e_{m}}|w_{e_{m}}^{c}\right)\mathrm{Pr}\left(w_{e_{m}}^{c}|a_{e_{m-1}}^{l}\right)\mathrm{Pr}\left(a_{e_{m-1}}^{l}|w_{e_{m-1}}^{c}\right)\times \\ \mathrm{Pr}\left({\tilde{s}}_{0}\right)\mathrm{Pr}\left({\tilde{x}}_{0}\right)\prod_{t_{i}=1}^{T_{i}}\mathrm{Pr}\left({\tilde{z}}_{e_{m},t_{i}}|{\tilde{x}}_{e_{m},t_{i}}\right)\mathrm{Pr}\left({\tilde{x}}_{e_{m},t_{i}}|{\tilde{s}}_{e_{m},t_{i}}\right)\mathrm{Pr}\left({\tilde{s}}_{e_{m},t_{i}}|a_{e_{m},t_{i-1}}^{p}\right)\mathrm{Pr}\left(a_{e_{m},t_{i-1}}^{p}|a_{e_{m},t_{i-2}}^{p},w_{e_{m-1}}^{c}\right). \end{array}$$

#### 4.3.1. Action Selection

The UAV performs two types of actions: one related to the targeted hotspot and the other pertaining to controlling its motion while moving towards it. To do this, the UAV relies on two AIn tables to select these actions. The first table encodes the relationship between the words and the discrete actions at the event scale defined as:

(21) $${\mathrm{AIn}}_{1}=\left[\begin{array}{cccc} \mathrm{Pr}(a_{1}^{l}|w_{1}^{c}) & \mathrm{Pr}(a_{2}^{l}|w_{1}^{c}) & \ldots & \mathrm{Pr}(a_{U}^{l}|w_{1}^{c})\\ \mathrm{Pr}(a_{1}^{l}|w_{2}^{c}) & \mathrm{Pr}(a_{2}^{l}|w_{2}^{c}) & \ldots & \mathrm{Pr}(a_{U}^{l}|w_{2}^{c})\\ \vdots & \vdots & \vdots & \vdots \\ \mathrm{Pr}(a_{1}^{l}|w_{C}^{c}) & \mathrm{Pr}(a_{2}^{l}|w_{C}^{c}) & \ldots & \mathrm{Pr}(a_{U}^{l}|w_{C}^{c}) \end{array}\right],$$

where $0\leq \mathrm{Pr}(a_{i}^{l}|w_{j}^{c})\leq 1$ and $\sum_{j=1}^{J}\mathrm{Pr}\left(a_{i}^{l}|w_{j}^{c}\right)=1,\forall j$. The other table encodes the relationship between the words and the continuous actions at the slot scale:

(22) $${\mathrm{AIn}}_{2}=\left[\begin{array}{cccc} \mathrm{Pr}(a_{1}^{p}|w_{1}^{c}) & \mathrm{Pr}(a_{2}^{p}|w_{1}^{c}) & \ldots & \mathrm{Pr}(a_{U}^{p}|w_{1}^{c})\\ \mathrm{Pr}(a_{1}^{p}|w_{2}^{c}) & \mathrm{Pr}(a_{2}^{p}|w_{2}^{c}) & \ldots & \mathrm{Pr}(a_{U}^{p}|w_{2}^{c})\\ \vdots & \vdots & \vdots & \vdots \\ \mathrm{Pr}(a_{1}^{p}|w_{C}^{c}) & \mathrm{Pr}(a_{2}^{p}|w_{C}^{c}) & \ldots & \mathrm{Pr}(a_{U}^{p}|w_{C}^{c}) \end{array}\right],$$

where $0\leq \mathrm{Pr}(a_{i}^{p}|w_{j}^{c})\leq 1$ and $\sum_{j=1}^{J}\mathrm{Pr}\left(a_{i}^{p}|w_{j}^{c}\right)=1,\forall j$.

The decisions made by the UAV to select actions that represent the targeted hotspot depend on the current word (i.e., the current location of the UAV), which is determined by the probability entries in (21). Thus, discrete actions are sampled from:

(23) $$a_{e_{m}}^{l}∼\mathrm{Pr}\left(.|w_{e_{m}}^{c}\right),$$

where $a_{e_{m}}^{l}$ is the selected discrete action at event $e_{m}$ that impacts future environmental hidden states and observations at event $e_{m+1}$. This ensures that the decisions made by the UAV are targeted towards the desired hotspots. Once the targeted hotspot is chosen (i.e., $a_{e_{m}}^{l}$), the UAV will then select a second action ($a_{e_{m}}^{p}$) that dictates how it will reach the targeted hotspot. This action is determined by the UAV’s starting hotspot and UAV’s target (represented by word $w_{e_{m}}^{c}$) and involves a series of actions at a more detailed time scale (slot scale). At the beginning of event $e_{m}$, the UAV selects the initial continuous action at the initial time slot $t_{1}$ of that event according to:

(24) $$a_{e_{m},t_{1}}^{p}=\mathrm{randint}(1,|A^{p}\left|\right),$$

where $A^{p}=\left\{North,South,East,West\right\}$, $|A^{p}|$ is the total number of available predefined actions, and $\mathrm{randint}(1,|A^{p}|)$ is a function representing a uniform distribution that generates an integer uniformly between 1 and $|A^{p}|$. During event $e_{m}$, the following continuous actions in the subsequent time slots $t_{i}$ are chosen based on previous continuous actions and prediction errors. More details on this will be explained later.

#### 4.3.2. Prediction and Perception

The UAV can anticipate the outcomes of joint actions at different time scales and levels of hierarchy. On a long-term scale, the UAV expects an increase in the number of served users after each event and every discrete action representing the targeted hotspots. This helps the UAV achieve its primary goal. On a smaller scale, while moving towards the targeted hotspot, the UAV anticipates reaching its second goal with each continuous action it takes during each time slot. Hence, the predictions are performed at two different temporal scales.

At the event scale, to predict the coupling word $w_{T,e_{m}}^{c}$, UAV employs a particle filter (PF) that propagates a set ${\left\{w_{T,e_{m}}^{c(n)},\omega_{T,e_{m}}^{l(n)}\right\}}_{n=1}^{N}$ of equally weighted particles sampled from the matrix $\Pi_{w^{c}}$ defined in (19). The UAV expresses its belief of how a specific word changes into another based on the performed action through a probabilistic form $\mathrm{Pr}(w_{T,e_{m}}^{c(n)}|w_{T,e_{m-1}}^{c(n)},a_{T,e_{m-1}}^{l})$. The predicted coupled word comprises the predicted generalized letter (${\tilde{l}}_{T,e_{m}}^{\left(n\right)}$) and predicted token ($\Theta_{T,e_{m}}^{\left(n\right)}$) since the word is formed by coupling these two components. For each propagated particle, UAV employs a Kalman filter (KF) to predict the continuous state ${\tilde{x}}_{T,e_{m}}^{l(n)}$ explaining the dynamics of the data rate. The KF relies on the dynamic model defined in (15c), which can be represented by the probability distribution $\mathrm{Pr}({\tilde{x}}_{T,e_{m}}^{l(n)}|{\tilde{x}}_{T,e_{m-1}}^{l(n)},{\tilde{l}}_{T,e_{m}}^{\left(n\right)})$. The posterior refers to the updated belief that forms after considering previous observations. It is connected to predictions and can be expressed as follows: $\pi \left({\tilde{x}}_{T,e_{m}}^{l}\right)=\mathrm{Pr}\left({\tilde{x}}_{T,e_{m}}^{l(n)},{\tilde{l}}_{T,e_{m}}^{\left(n\right)}|{\tilde{z}}_{T,e_{m-1}}^{l}\right)$. As the UAV obtains new observations, diagnostic messages propagating in a bottom–up manner can be used to update the posterior according to:

(25) $$\pi \left({\tilde{x}}_{T,e_{m}}^{l}\right)=\pi \left({\tilde{x}}_{T,e_{m}}^{l}\right)\times \lambda \left({\tilde{x}}_{T,e_{m}}^{l}\right),$$

where $\lambda \left({\tilde{x}}_{T,e_{m}}^{l}\right)=\mathrm{Pr}\left(z_{T,e_{m}}^{l}|{\tilde{x}}_{T,e_{m}}^{l}\right)$. Likewise, particles weights are updated at the higher level following:

(26) $$\omega_{T,e_{m}}^{l(n)}=\omega_{T,e_{m}}^{l(n)}\times \lambda \left({\tilde{l}}_{T,e_{m}}\right),$$

where:

(27) $$\lambda \left({\tilde{l}}_{T,e_{m}}\right)=\lambda \left({\tilde{x}}_{T,e_{m}}^{l}\right)\mathrm{Pr}\left({\tilde{x}}_{T,e_{m}}^{l}|{\tilde{l}}_{T,e_{m}}\right)=\mathrm{Pr}\left(z_{T,e_{m}}^{l}|{\tilde{x}}_{T,e_{m}}^{l}\right)\mathrm{Pr}\left({\tilde{x}}_{T,e_{m}}^{l}|{\tilde{l}}_{T,e_{m}}\right),$$

and $\mathrm{Pr}\left({\tilde{x}}_{T,e_{m}}^{l}|{\tilde{l}}_{T,e_{m}}\right)∼N\left(\mu_{{\tilde{l}}_{T,e_{m}}},\sigma_{{\tilde{l}}_{T,e_{m}}}\right)$.

On the other hand, at the slot scale, the UAV predicts the consequence of the continuous actions following the same approach explained earlier. By employing another PF, the UAV can predict the evolution of the discrete states $s_{e_{m},t_{i}}$ realizing the discrete zone of the UAV’s trajectory forming a token $\Theta_{e_{m}}$. The UAV believes that the discrete states evolve in accordance with $\mathrm{Pr}(s_{e_{m,t_{i}}}|s_{e_{m,t_{i-1}}},\Theta_{e_{m}},a_{e_{m},t_{i-1}}^{p})$. The PF propagates a set of particles representing the predicted discrete states, ${\left\{s_{e_{m},t_{i}}^{\left(n\right)},\omega_{e_{m},t_{i}}^{\left(n\right)}\right\}}_{n=1}^{N}$, that are sampled using the transition matrix $\Pi_{\Theta }$ defined in (14). Consequently, a bank of KFs is employed to predict the continuous states representing the UAV’s positions using the dynamic model defined in (15d), which can be expressed as $\mathrm{Pr}({\tilde{x}}_{e_{m},t_{i}}|{\tilde{x}}_{e_{m},t_{i-1}},s_{e_{m},t_{i}}^{\left(n\right)})$. The posterior associated with the predicted states is given by:

(28) $$\pi \left({\tilde{x}}_{e_{m},t_{i}}\right)=\mathrm{Pr}\left({\tilde{x}}_{e_{m},t_{i}}^{\left(n\right)},s_{e_{m},t_{i}}^{\left(n\right)}|{\tilde{z}}_{e_{m},t_{i-1}}\right)=\int \mathrm{Pr}\left({\tilde{x}}_{e_{m},t_{i}}|{\tilde{x}}_{e_{m},t_{i-1}},s_{e_{m},t_{i}}^{\left(n\right)}\right)\lambda \left({\tilde{x}}_{e_{m},t_{i-1}}^{\left(n\right)}\right)d{\tilde{x}}_{e_{m},t_{i-1}},$$

where $\lambda \left({\tilde{x}}_{e_{m},t_{i-1}}^{\left(n\right)}\right)=\mathrm{Pr}\left({\tilde{z}}_{e_{m},t_{i-1}}|{\tilde{x}}_{e_{m},t_{i-1}}\right)$ is the diagnostic message propagated in a bottom–up manner after observing ${\tilde{z}}_{e_{m},t_{i-1}}$ at time slot $t_{i-1}$. When a new observation is received, diagnostic messages can be utilized to update the UAV’s belief in hidden states. The belief in continuous states can be corrected by updating the posterior using:

(29) $$\pi \left({\tilde{x}}_{e_{m},t_{i}}\right)=\pi \left({\tilde{x}}_{e_{m},t_{i}}\right)\times \lambda \left({\tilde{x}}_{e_{m},t_{i}}^{\left(n\right)}\right).$$

Meanwhile, the belief in discrete states can be updated by adjusting the weights of the particles following:

(30) $$\omega_{e_{m},t_{i}}^{\left(n\right)}=\omega_{e_{m},t_{i}}^{\left(n\right)}\times \lambda \left({\tilde{s}}_{e_{m},t_{i}}\right),$$

where $\lambda \left({\tilde{s}}_{e_{m},t_{i}}\right)=\lambda \left({\tilde{x}}_{e_{m},t_{i}}\right)\mathrm{Pr}\left({\tilde{x}}_{e_{m},t_{i}}|s_{e_{m},t_{i}}\right)$.

#### 4.3.3. Abnormality Measures and Action Update

At each level of the hierarchy, the messages that predict what should happen are compared to the sensory messages that report what is actually happening. This comparison results in several indicators of abnormalities and prediction errors. We can determine how well the current observations match the model’s predictions by examining these indicators at each level. Additionally, we can use the prediction errors to figure out how to prevent these abnormalities from occurring in the future. The observations of the UAV are influenced by its actions. Thus, if an abnormality is detected, it means that the actions taken were incorrect. The UAV can use the prediction errors to make necessary corrections and prevent abnormalities in the future.

The UAV has the capability to evaluate ongoing actions by utilizing an abnormality indicator that calculates the difference between predicted states and observations. This is achieved through the calculation of the Bhattacharyya distance as follows:

(31) $$\Upsilon_{{\tilde{x}}_{e_{m},t_{i}}}=-ln\left(\mathrm{BC}\left(\pi \left({\tilde{x}}_{e_{m},t_{i}}\right),\lambda \left({\tilde{x}}_{e_{m},t_{i}}^{\left(n\right)}\right)\right)\right)=-ln\int \sqrt{\pi \left({\tilde{x}}_{e_{m},t_{i}}\right)\lambda \left({\tilde{x}}_{e_{m},t_{i}}^{\left(n\right)}\right)}d{\tilde{x}}_{e_{m},t_{i}},$$

where BC is the Bhattacharyya coefficient. It is to note that during exploration, the UAV’s expected states realize the target position, while during exploitation, the UAV’s expected states are guided by the tokens.

The abnormality indicator defined in (31) is associated with prediction errors calculated as:

(32) $$E_{{\tilde{x}}_{e_{m},t_{i}}}=\left[{\tilde{x}}_{e_{m},t_{i}},{\dot{E}}_{{\tilde{x}}_{e_{m},t_{i}}}\right]=\left[{\tilde{x}}_{e_{m},t_{i}},H^{-1}E_{{\tilde{z}}_{e_{m},t_{i}}}\right],$$

where $E_{{\tilde{z}}_{e_{m},t_{i}}}∼N\left(\mu_{E_{{\tilde{z}}_{e_{m},t_{i}}}},\Sigma_{E_{{\tilde{z}}_{e_{m},t_{i}}}}\right)$ depicts the prediction errors computed in the observation space, which is characterized by the following statistical properties:

(33a) $$\begin{array}{c} {\tilde{\mu }}_{E_{{\tilde{z}}_{e_{m},t_{i}}}}={\tilde{z}}_{e_{m},t_{i}}-H{\tilde{x}}_{e_{m},t_{i}}, \end{array}$$

(33b) $$\begin{array}{c} \Sigma_{E_{{\tilde{z}}_{e_{m},t_{i}}}}=H\Sigma_{E_{{\tilde{z}}_{e_{m},t_{i}}}}H^{⊺}+R, \end{array}$$

where (33a) is the Kalman innovation and (33b) is the innovation covariance.

In case the UAV encounters abnormal situations, it can use prediction errors to rectify its previous actions through first-order Euler integration following:

(34) $$a_{e_{m},t_{i}}^{p}=a_{e_{m},t_{i-1}}^{p}+\Delta_{t_{i}}{\dot{\mu }}_{{\tilde{x}}_{e_{m},t_{i}}},$$

where $\Delta_{t_{i}}$ is the step size.

On the other hand, the UAV can assess the discrete actions representing the targeted hotspots only after completing a full mission that includes a sequence of events. This is because the UAV needs to determine if the selected hotspots were efficiently reached in their designated order to achieve the intended goal of maximizing the sum rate. As previously stated, a series of actions (or generalized letters) form a word, and the UAV checks whether the resulting word fulfills the intended goal. Therefore, to evaluate the formed word, it is necessary to consider the cumulative abnormality indicator. This indicator adds up the abnormalities that measure the divergence between what was expected and what was observed at each event. The abnormality indicator itself is defined as:

(35) $$\Upsilon_{{\tilde{x}}_{T,e_{m}}}=-ln\left(\mathrm{BC}\left(\pi \left({\tilde{x}}_{T,e_{m}}\right),\lambda \left({\tilde{z}}_{T,e_{m}}\right)\right)\right)=-ln\int \sqrt{\pi \left({\tilde{x}}_{T,e_{m}}\right)\lambda ({\tilde{z}}_{T,e_{m}}})d{\tilde{x}}_{T,e_{m}}.$$

while the cumulative abnormality indicator is defined as follows:

(36) $$\Upsilon_{{\tilde{x}}_{T}}=\sum_{e_{m}=1}^{E}\Upsilon_{{\tilde{x}}_{T,e_{m}}},$$

where *E* is the total number of events that occur during the flight mission, which lasts for a duration of *T*.

In case the UAV detects a high cumulative abnormality, this indicates that the entire mission was unsuccessful. In this case, the UAV must correct the action selection process by updating its strategy of forming the word. This can be done by updating the active inference table defined in (21) as follows:

(37) $$\mathrm{Pr}\left(a_{e_{m}}^{l}|w_{e_{m}}^{c}\right)=\mathrm{Pr}\left(a_{e_{m}}^{l}|w_{e_{m}}^{c}\right)-\gamma ,$$

where the gradient γ determines the amount by which the probability should be decreased.

Additionally, if the mission is successful with minimal abnormalities, the transition matrix specified in (12) will be modified as follows:

(38) $$\mathrm{Pr}\left({\tilde{l}}_{i}|{\tilde{l}}_{j}\right)=\mathrm{Pr}\left({\tilde{l}}_{i}|{\tilde{l}}_{j}\right)+\bar{\gamma },$$

where *i* and *j* are part of the successful word representing the sequence of hotspots visited by the UAU during its successful mission and $\bar{\gamma }$ is the gradient that determines the amount by which the probability should be increased.

## 5. Numerical Results and Discussion

In this section, we will thoroughly assess how well the proposed framework performs in designing a trajectory for the UAV that effectively allows it to attain the highest total sum-rate possible with the cell. In our simulations, we examined a situation where a single UAV is providing service to several users who are located in different hotspots across a square geographic area of 1000×1000 $m^{2}$. The main simulation parameters are listed in Table 2. It is assumed that the altitude of the UAV remains constant at $z_{u}=100$ m [65]. Throughout the training process, we place a total of N=80 hotspots in various random locations across the geographical area. The frequency of user presence and requests within each hotspot adheres to the Poisson distribution. We generated a training set D that consists of *M* examples corresponding to different realizations. Each realization (*m*) consists of seven hotspots picked randomly from the *N* total hotspots, and the users’ requests in each hotspot were generated following Poisson distribution. The TSPWP method was used to solve the *M* examples in D, generating *M* trajectories (TSPWP instances) and *M* sequences of the order in which the hotspots are visited, which were saved in $L^{+}$ and $Q^{+}$, respectively.

**Figure 8:**
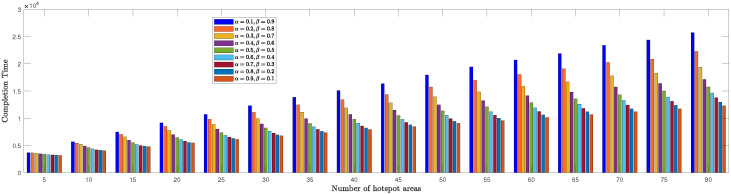
TSPWP’s completion time performance for varying alpha and beta values, as well as changes in the number of hotspots.

**Figure 9:**
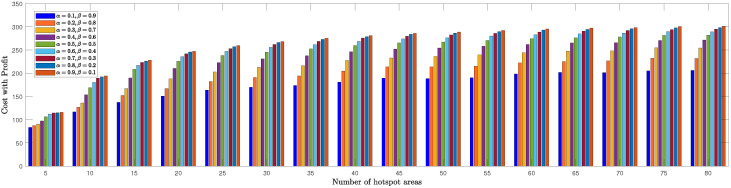
TSPWP’s cost with profit performance for varying alpha and beta values, as well as changes in the number of hotspots.

We evaluated the TSPWP performance by conducting a thorough analysis of completion time and cost with profit metrics for different numbers of hotspots to determine the optimal α and β values mentioned in (6a). In Figure 8, we see how the completion time of TSPWP was impacted by various α and β values, as well as changes in the number of hotspots. Meanwhile, Figure 9 displays the TSPWP performance in terms of cost with profit for different α and β settings while also altering the number of hotspots. It is evident from Figure 8 that the completion time increases as the number of hotspots increases, as having more hotspots makes the trajectory longer. It is worth noting that the cost with profit rose gradually as the number of hotspots increased, especially between five and twenty, as shown in Figure 9. However, after twenty hotspots, the cost with profit slightly rose due to the reduction of profit (i.e., the accumulated sum-rate) from the cost (i.e., the traveling distance between the hotspots). This effect became stable for higher hotspots and had a minimal impact on the overall cost with profit. By analyzing the data, we have found that the ideal α and β values for achieving both minimal completion time and maximum profit with cost are 0.9 and 0.1, respectively. Therefore, we will use these values when implementing TSPWP with 2-OPT.

To solve each realization *m*, we used the TSPWP with α=0.9 and β=0.1, as previously mentioned. The TSPWP with 2-OPT gave us the solution (i.e., the TSPWP instance), which includes the trajectory and the order of the hotspots to visit. We then created two sub-dictionaries from the *M* TSPWP instances. The first sub-dictionary comprised all the words that made up the TSPWP trajectories, which use letters to represent the hotspots (explained in Section 4.2.1). The second sub-dictionary contained all the tokens that showed the path between two adjacent letters (hotspots), as described in Section 4.2.1.

**Figure 10:**
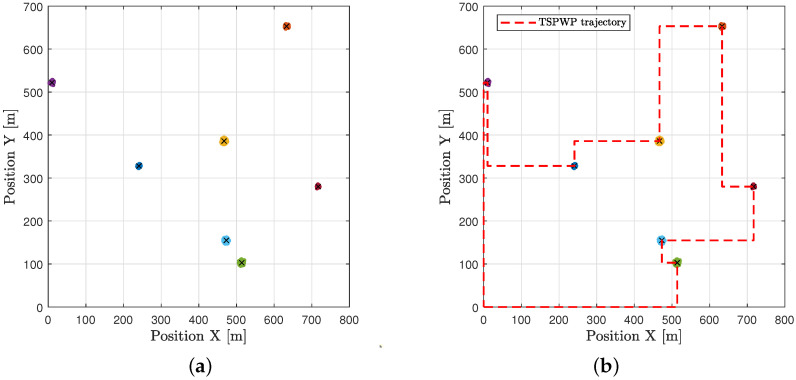
An example of one realization: (**a**) Seven hotspots scattered randomly across the geographical area labeled with different letters, and each has a varying number of active users requesting service. (**b**) The trajectory provided by the TSPWP.

**Figure 11:**
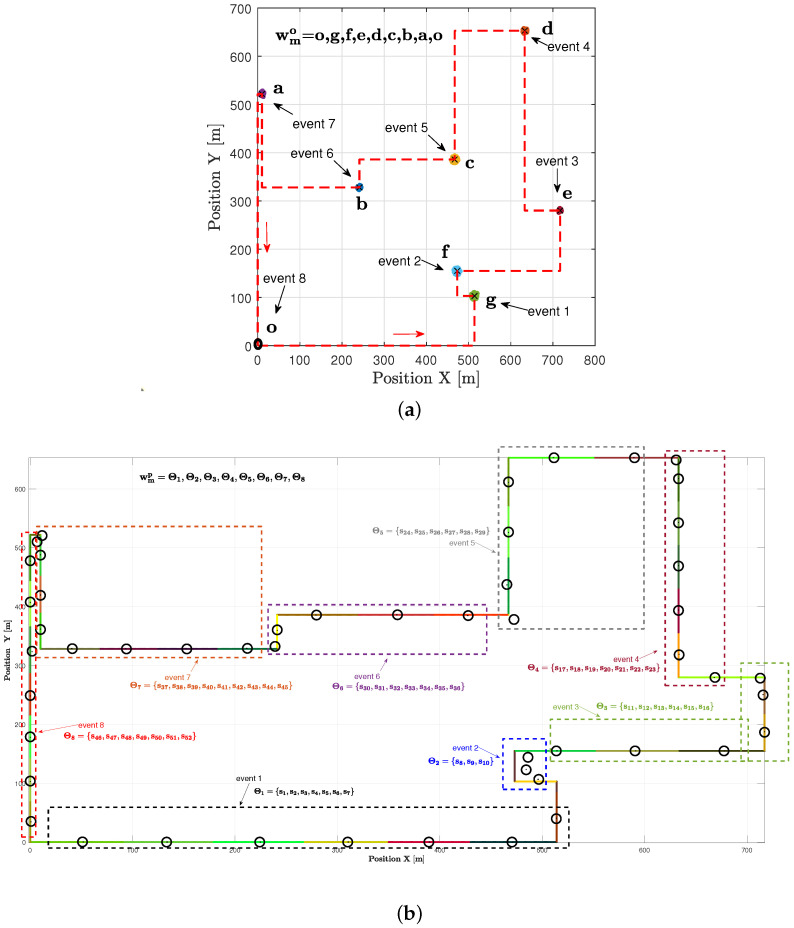
The process of forming the dictionary: (**a**) The events that have been occurred during the flight and the generated word consisting of the letters visited by the UAV. Event 1 occurs after reaching letter g starting from letter o. Event 2 occurs after reaching letter f from g. Event 3 occurs after reaching letter e from f. Event 4 occurs after reaching letter d from e. Event 5 occurs after reaching letter c from d. Event 6 occurs after reaching letter b from c. Event 7 occurs after reaching letter a from b. Event 8 occurs after returning to the origin from a. (**b**) The clusters obtained after clustering the trajectory. Clusters are labeled as letters. The generated tokens each consist of several letters corresponding to a specific event and thus explaining the path to follow between two adjacent letters.

In the example shown in Figure 10a, there is one realization with seven hotspots scattered randomly in the geographic area. Each hotspot has some active users who need resources. The goal is to start from the initial station at the origin, visit each hotspot only once, serve the users there, and then return to the origin within a specific time frame. The realization depicted in Figure 10a is used as input to the TSPWP with 2-OPT method. Latter will produce the TSPWP instance, which includes the trajectory and the order of visited hotspots, as demonstrated in Figure 10b. To create the global dictionary, TSPWP instances from *M* examples are utilized, which include sub-dictionary 1 and sub-dictionary 2. Sub-dictionary 1 records the events that take place during the flight mission, such as when the UAV reaches hotspot *j* after departing from hotspot *i*. The process of detecting different events and forming a word representing the sequence of hotspots served during a flight mission is illustrated in Figure 11a. In this process, hotspots are considered as letters, and the full trajectory represents a word. The first event occurs after reaching the letter “g” starting from “o”. The second event occurs after reaching “f” from “g”, and so on for the third and subsequent events. The final event occurs when the UAV returns to the initial location, represented by the letter “o”, starting from “a”. Therefore, the word describing the mission is defined as “w = o, g, f, e, d, c, b, a, o”. By contrast, if we cluster the trajectory data (which include positions and velocities), we can see the resulting clusters in Figure 11b. Each event that was previously detected will be linked to the set of clusters that form the path from one letter to another, as illustrated in Figure 11b. A token is created for each event, and all the tokens are combined to form the resulting word, which represents the path followed during the mission. Throughout the training process, the same procedure is carried out for *M* examples in order to create the words that indicate the sequence of targeted hotspots and the words that describe the movement from one hotspot to the next. These two sets of words are coupled statistically to create a world model that the UAV will use during the active inference (testing) process to plan a suitable trajectory based on encountered situations (realizations).

**Figure 12:**
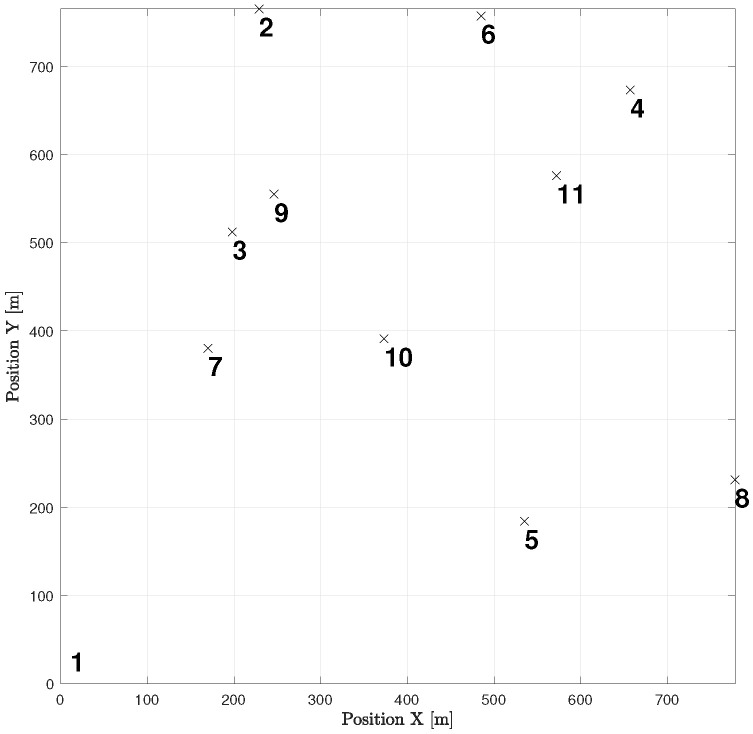
An example of a testing realization including 11 hotspots.

**Figure 13:**
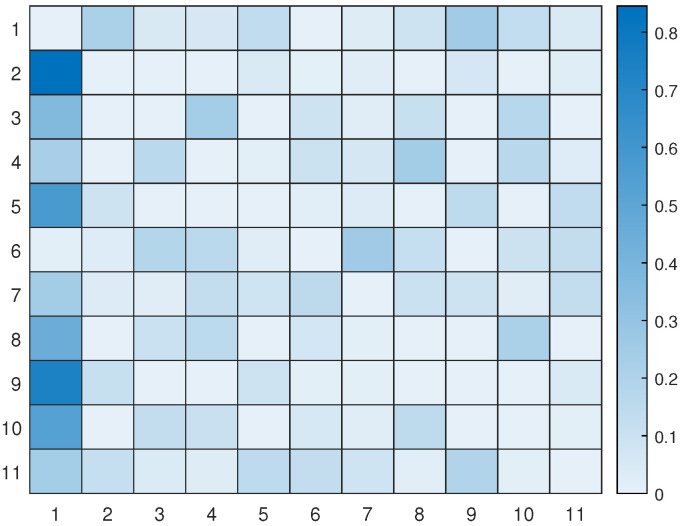
The transition matrix encoding the probabilities of passing from one letter to another based on the examples solved during training.

Let us take a look at how a UAV, using active inference, completes a mission. For instance, suppose there are 11 hotspots in a given testing scenario as shown in Figure 12. The UAV will rely on the world model, made up of two sub-dictionaries, that it learned during training to successfully navigate the testing scenario. First, the UAV examines the current letters and matches them against the words listed in sub-dictionary 1. This process helps to establish how closely they resemble each other in the current testing scenario. After that, the UAV chooses the closest word from the dictionary and uses it as a starting point to create the initial graph. The goal is to expand the graph by adding new letters to form a word that enables an efficient trajectory to reach all hotspots (letters) and serve their users as quickly as possible. To achieve this, one letter is added during each iteration, with the number of iterations depending on the size of the reference graph and the number of new letters required to include all available letters in the current configuration. To update the graph and make it directed, one link must be removed from the reference graph, and two links must be added to the newly added letter or node at every iteration. The transition matrix, which encodes the probabilistic relationships among the letters, is crucial at each step and can be found in Figure 13. This matrix determines whether it is possible to transition from a letter already present in the reference graph to the newly added letter. The transition matrix is learned after solving *M* examples during training and allows for the generation of words based on probability entries.

**Figure 14:**
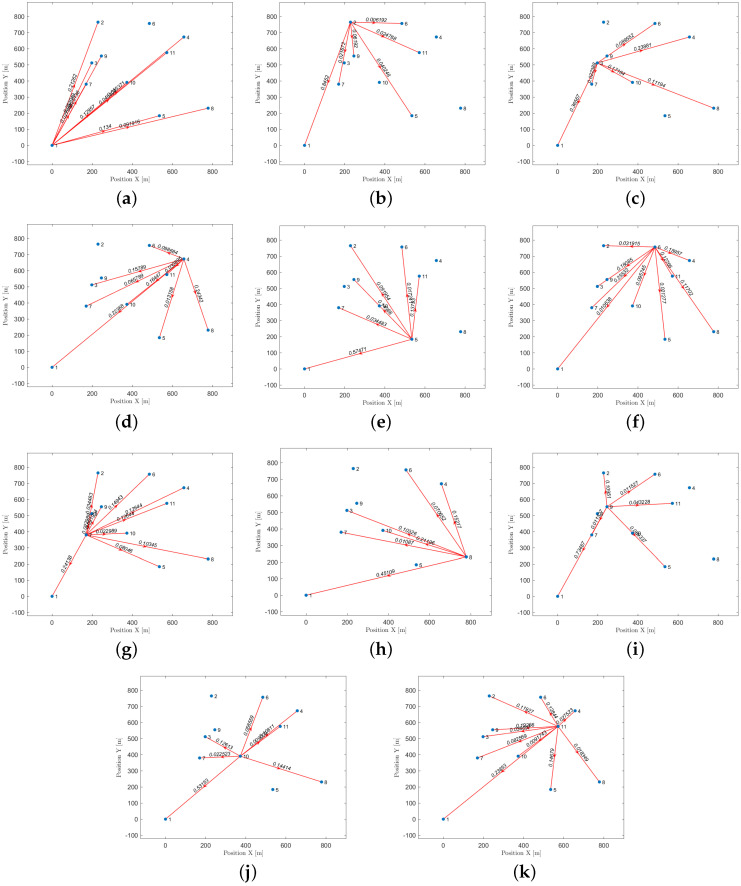
The transition probabilities suggested by the world model to generate a word that might solve the current realization: (**a**) Possible letters to target starting from letter 1. (**b**) Possible letters to target starting from letter 2. (**c**) Possible letters to target starting from letter 3. (**d**) Possible letters to target starting from letter 4. (**e**) Possible letters to target starting from letter 5. (**f**) Possible letters to target starting from letter 6. (**g**) Possible letters to target starting from letter 7. (**h**) Possible letters to target starting from letter 8. (**i**) Possible letters to target starting from letter 9. (**j**) Possible letters to target starting from letter 10. (**k**) Possible letters to target starting from letter 11.

Figure 14 displays all the available pathways from the 11 hotspots to other letters. Depending on the current letter, one can determine which letters are reachable. For instance, if one starts at letter 1 (the initial location), one cannot transition to letter 6, but one can transition to the other 9 letters with varying probabilities. Similarly, if one reaches letter 2, one cannot go towards letters 3, 4, 8, and 10, and so on. It is worth noting that the probability values provided by the world model prevent unnecessary transitions that will not help the UAV reach its desired goal.

**Figure 15:**
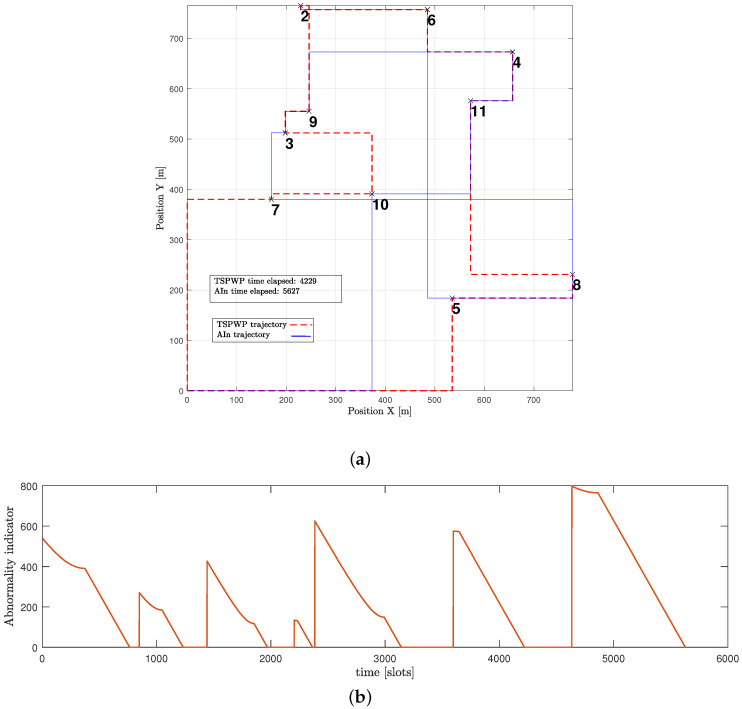
A word generated using active inference before convergence: (**a**) The trajectory followed by the UAV based on active inference before the convergence. (**b**) The abnormalities that occurred during the flight mission.

The example shown in Figure 15a expresses a word generated by the UAV through the proposed method but before it fully converged. The generated word is not optimal as it contains hotspots in the wrong order, which causes the mission to take longer and increases the time needed to return to the initial location. Furthermore, Figure 15b shows that the UAV detected abnormalities during most of the operation events. When the UAV detects abnormalities in its position, it is usually because it is not close enough to its goal. The UAV aims for a specific letter that represents its target. It is drawn towards that goal and then assesses its distance from the goal after each continuous action that represents its velocity. If there are any abnormalities, the UAV can use prediction errors to correct its actions and adjust its path to reach the targeted letter. For instance, during event 1, the UAV perceived high abnormalities and prediction errors while it was still far from the intended letter, with the starting letter being 1 and the targeted being 10. However, utilizing the prediction error, the UAV was able to adjust its actions and reach the destination faster. This resulted in the abnormality signals gradually decreasing until they reached zero, indicating that the UAV had indeed arrived at the targeted destination.

**Figure 16:**
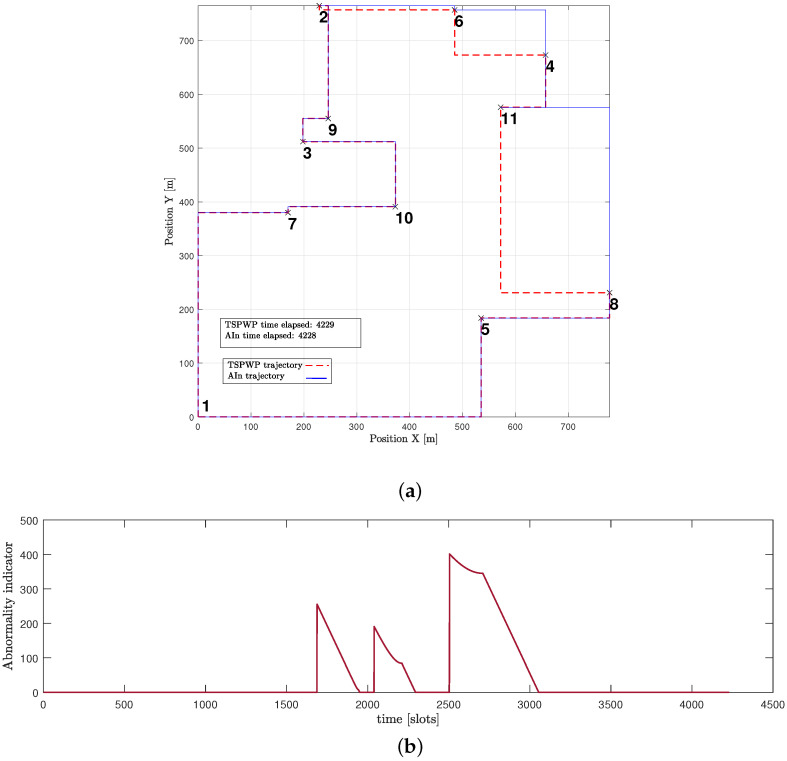
A word generated using active inference after convergence: (**a**) The trajectory followed by the UAV based on active inference before the convergence. (**b**) The abnormalities that occurred during the flight mission.

Figure 16a presents another example of a word created by the UAV after convergence. The proposed approach enabled the UAV to design a trajectory that is comparable to the one generated by the TSPWP with 2-OPT, with a similar completion time. It is noticeable that the UAV was successful in reducing high abnormalities in various events, as depicted in Figure 16b, compared to the example shown before convergence. This reduction is due to the UAV’s ability to differentiate between similar events encountered before and deduce the optimal path immediately.

**Figure 17:**
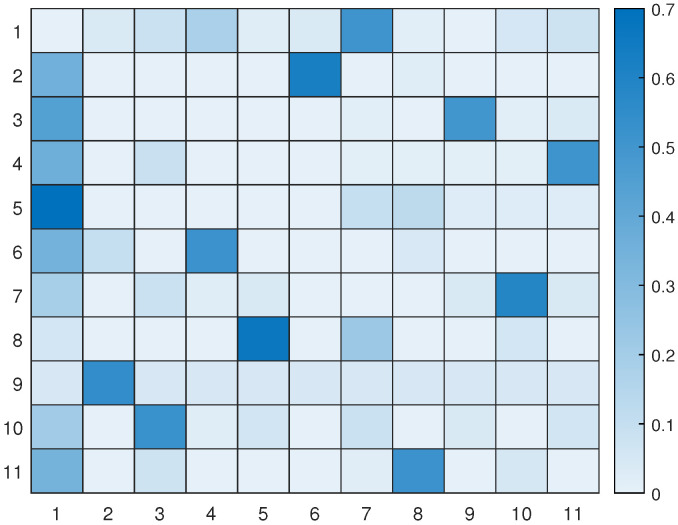
The updated transition matrix encoding the probabilities of passing from one letter to another after convergence to solve the example shown in Figure 12.

Figure 17 displays the updated transition matrix for 11 letters, which includes corrected probability entries detailing the possible transitions between the available letters. This updated transition matrix was rectified using the one exhibited in Figure 13.

**Figure 18:**
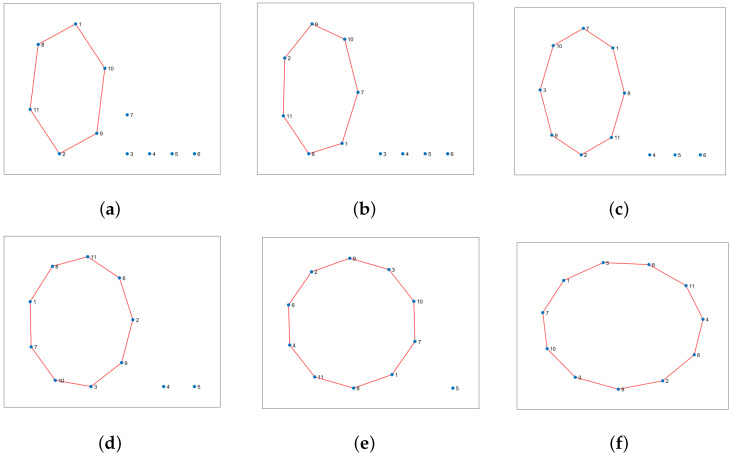
This is a graphic explanation of the process for creating new words from a base word found in the dictionary: (**a**) The reference word is represented graphically, and the new letters encountered in the new situation should be added to the reference graph. (**b**) The updated graph (word) after adding letter 7. (**c**) The updated graph (word) after adding letter 3. (**d**) The updated graph (word) after adding letter 6. (**e**) The updated graph (word) after adding letter 4. (**f**) The updated graph (word) after adding letter 5.

The process of creating new words is shown in Figure 18. The first step is to select a reference word from the dictionary by comparing the available letters in the current realization with the encoded words in the dictionary. The UAV selects the word with the highest probability of being a match based on the similarity of its letters to the available ones. The matching letters from the most similar word are then used as a reference for creating new words. This reference word is represented graphically as a closed loop, as demonstrated in Figure 18a. The initial graph is expanded by adding one letter at a time, as illustrated in the figure. This insertion approach dramatically reduces the likelihood of the UAV needing to determine the optimal visiting order. For instance, if there are 11 nodes to visit, and each node must be visited only once, there are approximately 11! (∼39 million) possible word combinations for which to find the correct order, which is a time-consuming and challenging task, particularly when using a trial-and-error method. However, the proposed word formation mechanism decreases the number of possible combinations from 11! to just 40. In Figure 18a, there are six potential ways to create a new word by adding the first letter to the reference graph. Figure 18b has seven possible words, while the other graphs feature eight, nine, and ten options. The total number of combinations is 40, which is calculated by adding the number of edges in each graph.

**Figure 19:**
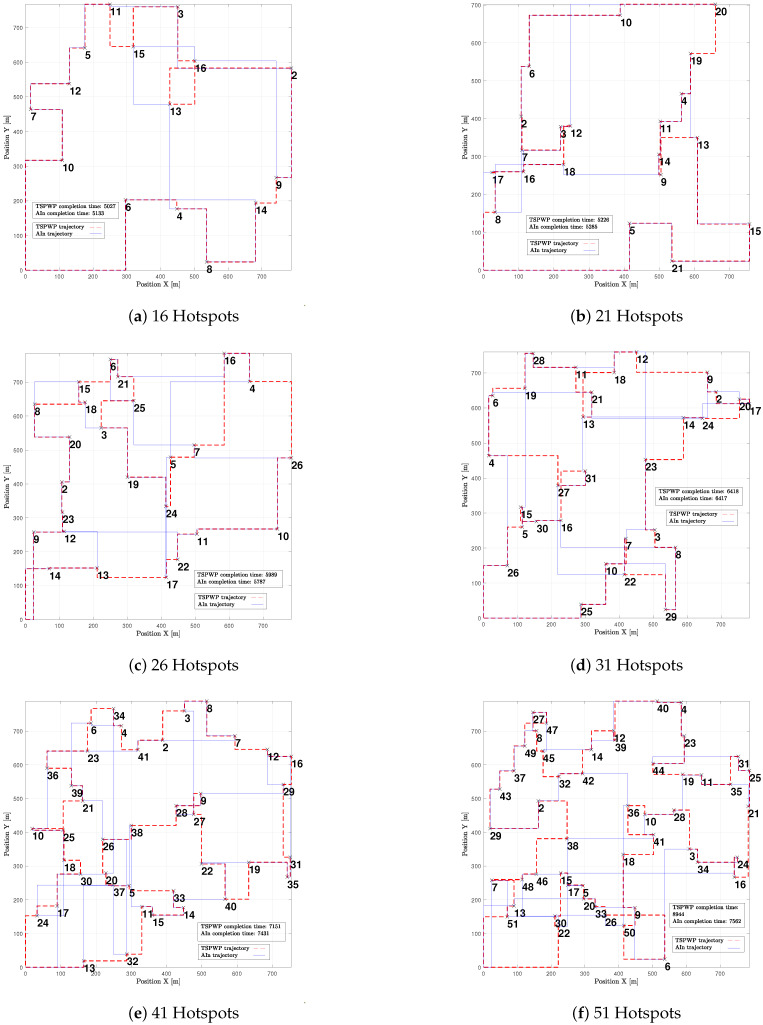
The figure displays various examples with varying numbers of hotspot areas, along with the solutions produced by the proposed method (AIn) and the TSPWP utilizing 2-OPT.

In Figure 19, one can see different examples with different numbers of hotspot areas. The trajectories generated by the proposed method (AIn) and the TSPWP using 2-OPT are also shown, along with their respective completion times. It is evident that the proposed approach produces alternative solutions when compared to the TSPWP with 2-OPT. In some cases, it also results in a quicker completion time as shown in Figure 19c,d,f. This highlights the adaptability of the proposed method in deriving reasonable solutions that surpass those of the TSPWP.

**Figure 20:**
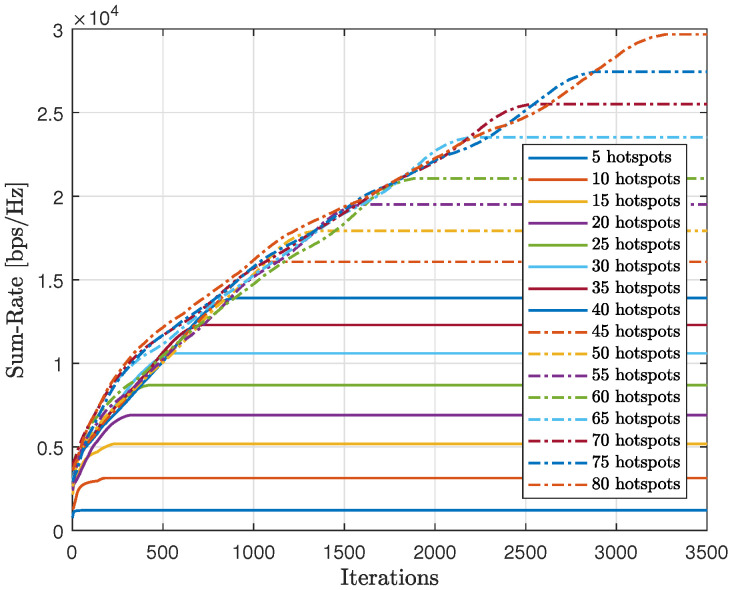
Convergence of the proposed approach (AIn) in terms of sum-rate for different numbers of hotspots.

**Figure 21:**
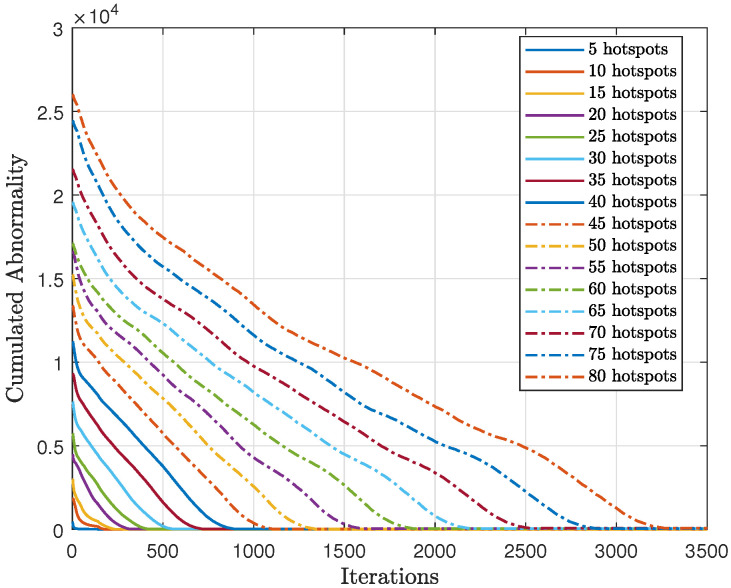
Cumulative abnormality convergence of the proposed approach (AIn) for different numbers of hotspots.

As shown in Figure 20, we tested the scalability of the proposed method (AIn) and compared the cumulative sum-rate convergence for various hotspots. We observed that as the number of hotspots increased, the cumulative sum-rate also increased. However, it took longer to find the best solution and reach convergence with more hotspots. This is because there were more possible generated words to test, which takes longer. By contrast, Figure 21 shows the cumulative abnormality for various numbers of hotspots. The trend of the cumulative abnormality is contrary to the cumulative sum-rate. It begins with high values and gradually decreases until reaching quasi-zero at convergence. As the number of hotspots increases, the time taken to reach quasi-zero abnormality also increases.

**Figure 22:**
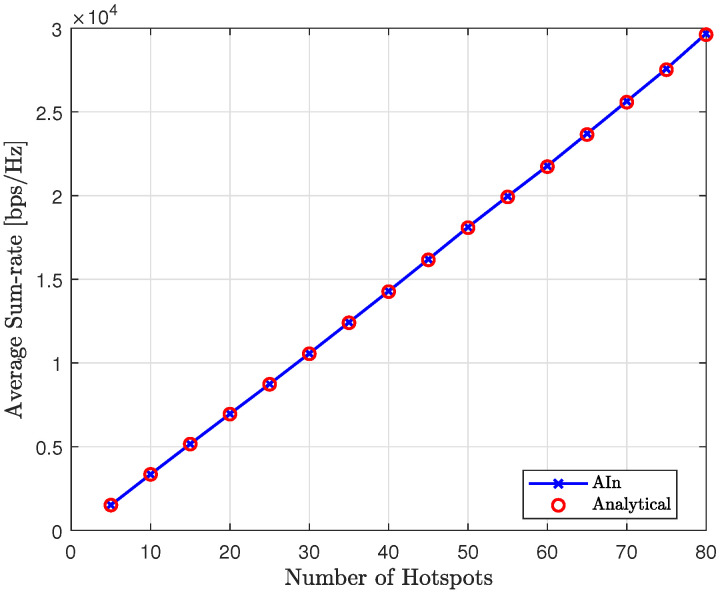
The average sum-rate of the proposed approach (AIn) compared to the analytical value for various number of hotspots.

In Figure 22, we can see the average sum-rate of the proposed method at convergence for various numbers of hotspots, compared to the analytical sum-rate. It is clear that the proposed approach achieves the expected analytical sum-rate after convergence, regardless of the number of hotspots.

### Comparison with Modified Q-Learning

In this section, we compare the performance of the proposed approach (AIn) with a modified version of the conventional Q-learning (QL) [66]. To ensure a fair comparison, the modified-QL follows the same logic as the proposed approach. Thus, the modified version uses two probabilistic q-tables—one for mapping discrete states (hotspots) to discrete actions (targeted letters) and another for mapping discrete environmental regions to continuous actions (velocity). Unlike traditional QL, the q-values in these tables are represented as probability entries that range between 0 and 1.

As in the proposed method, we can see that the discrete states stand for the letters, and the discrete environmental regions stand for the clusters. In addition, the available letters during a specific realization make up the discrete action space, while four continuous actions representing different directions (*Up*, *Down*, *Left*, *Right*) make up the continuous action space. The reward function in modified-QL was designed using the TSPWP instances. If the modified-QL behaves similarly to the TSPWP, it will receive a positive reward (+1). Otherwise, the reward is zero.

**Figure 23:**
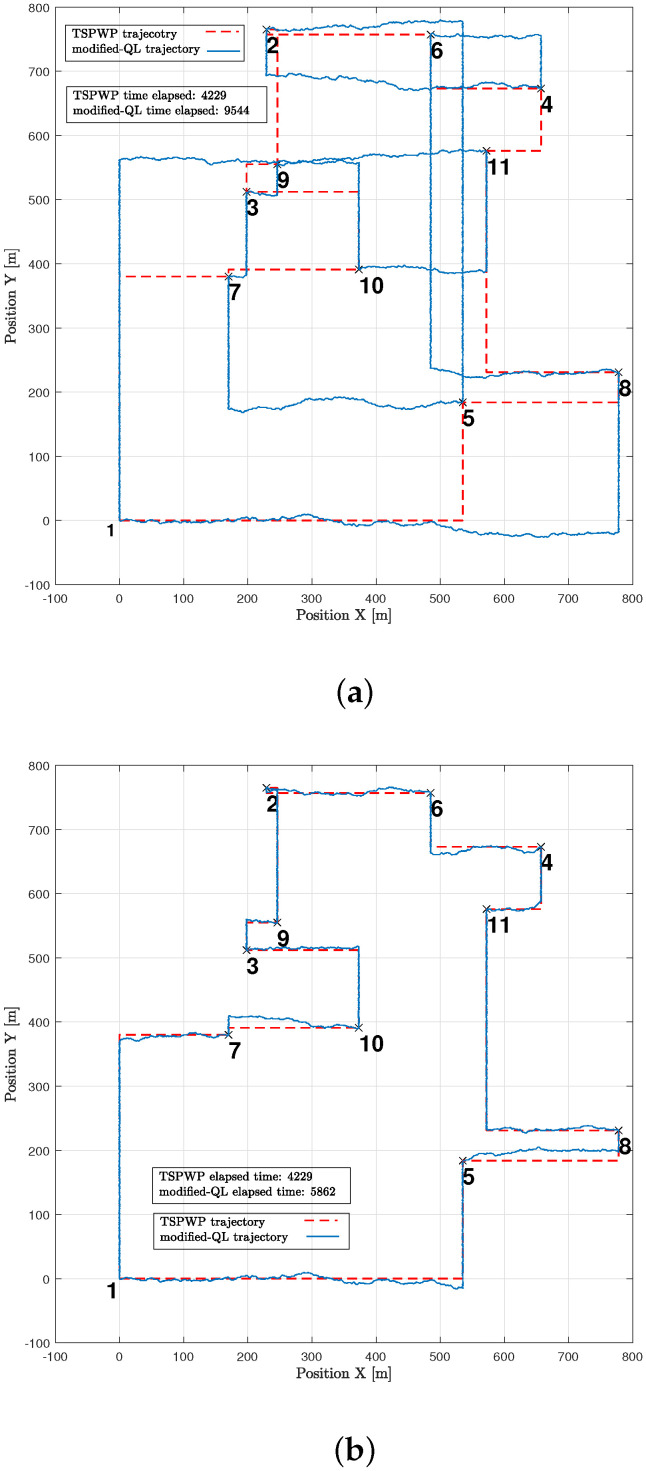
An example of the realization shown in Figure 12: (**a**) The trajectory followed by the UAV using the modified-QL before convergence. (**b**) The trajectory followed by the UAV using the modified-QL after convergence.

In Figure 23, an example similar to the one in Figure 10a is shown to illustrate how the modified-QL algorithm solved the mission both before and after convergence. Prior to convergence (Figure 23a), the modified-QL selected the wrong order of letters to visit, leading to a longer completion time. However, after convergence (Figure 23b), the algorithm discovered the correct order of letters, resulting in a reduced completion time, although it still fell short of the completion time achieved by the TSPWP with 2-OPT due to a slight deviation from the correct path. It is important to note that the agent’s movement was limited to traveling between two boundaries to simplify the process, which reduced the environmental states it could discover. Consequently, the modified-QL agent’s movements were guided by the TSPWP through positive and zero rewards.

**Figure 24:**
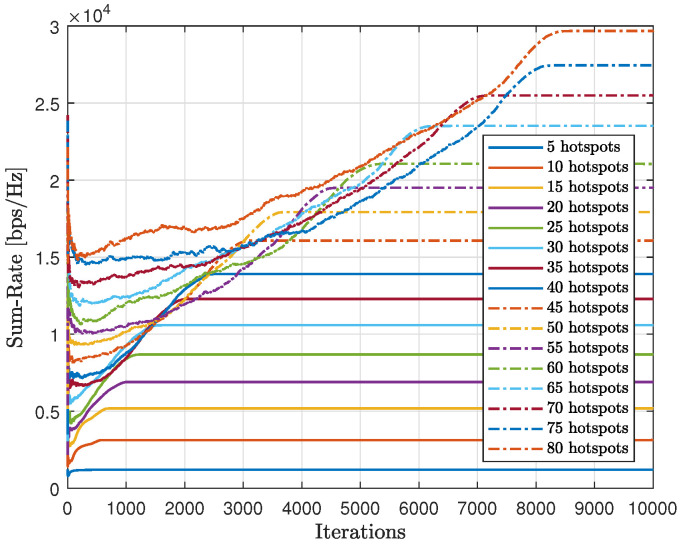
Convergence of the modified-QL in terms of sum-rate for different numbers of hotspots.

Figure 24 displays the gathered sum-rate in relation to the number of iterations, providing insight into the modified-QL’s overall performance and scalability with varying numbers of hotspots. It is clear that as the number of hotspots increases, both the collected sum-rate and the time to converge will also increase with the modified-QL. Despite requiring more iterations, the modified-QL achieved the same sum-rate at convergence as the proposed method.

**Figure 25:**
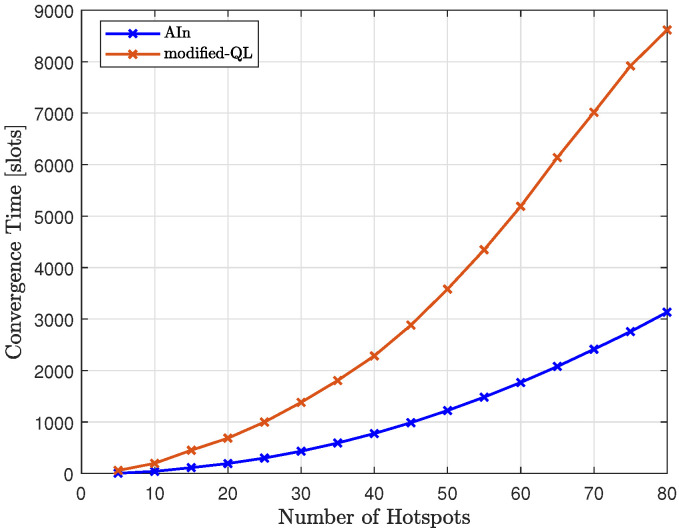
The convergence time of the proposed approach (AIn) compared to the convergence time of the modified-QL for different numbers of hotspots.

In Figure 25, we compared the convergence time of the proposed method (AIn) to that of the modified-QL, as we varied the number of hotspots. The results show that the proposed method requires less time to converge than the modified-QL. This difference was more noticeable as we increased the number of hotspots, with the gap between the two trends increasing. The modified-QL took longer to converge as we increased the number of hotspots, and it did so at a faster rate than AIn due to its random nature, which led to a higher number of possible words to try compared to AIn.

**Figure 26:**
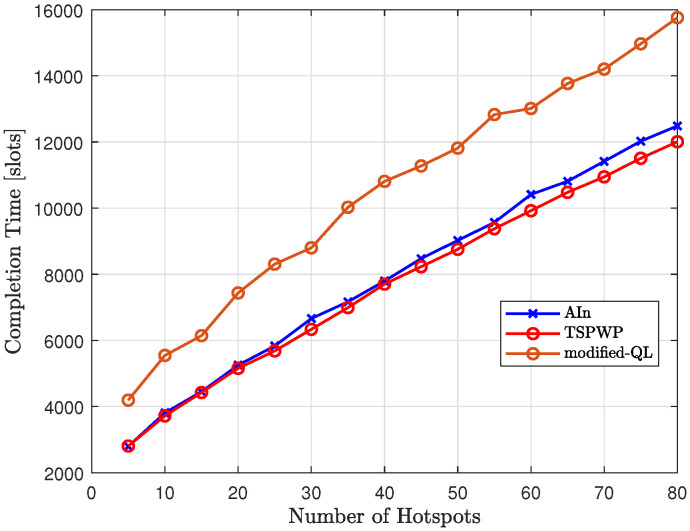
The performance of the proposed approach (AIn) in terms of completion time after convergence compared with TSPWP for different numbers of hotspots.

Figure 26 compares the completion time of our proposed method, AIn, to that of modified-QL and TSPWP with 2-OPT as the number of hotspots varies. The results show that modified-QL took longer to complete the missions due to slight deviations from the reference trajectories designed by TSPWP. These deviations were caused by the random actions performed before the convergence. On the other hand, AIn is able to complete missions faster than modified-QL thanks to its ability to deduce certain paths based on the world model and calculate prediction errors to correct continuous actions. This allows AIn to reach the target destination more quickly.

## 6. Conclusions and Future Directions

This paper studied the trajectory design problem in UAV-assisted wireless networks. In the considered system, a single UAV provides on-demand uplink communication service to ground users by flying around the environment. To solve this problem, we have proposed a goal-directed method based on active inference, consisting of two computation units. The first unit builds a world model to understand the surrounding environment, while the second unit makes decisions to minimize a cost function and achieve preferred outcomes. The world model represents a global dictionary that has been learned from instances generated by the TSPWP using a 2-OPT algorithm to solve various offline examples. The dictionary includes letters for hotspots, tokens for local paths, and words for complete trajectories and order of hotspots. By analyzing the dictionary, we can understand the decision maker’s grammar, specifically the TSPWP strategy, and how it utilizes the available letters to form tokens and words. To accurately represent the properties of TSPWP graphs at different levels of abstraction and time scales, we developed a novel hierarchical representation called the coupled multi-scale generalized dynamic Bayesian network (C-MGDBN) that structures the gathered knowledge (i.e., the global dictionary).

Simulation results indicate that the proposed method performs better than the traditional Q-learning algorithm. It provides quick, stable, and alternative solutions with good generalization capabilities. Additionally, the results demonstrate that our approach can be scaled up to larger instances, despite being trained on smaller ones, proving its effectiveness in generalization. Furthermore, we have proven that our method can solve a complex problem (known as NP-hard) by significantly reducing the number of actions the UAV needs to take to solve a specific example.

In future work, we plan to tackle the challenge of determining the optimal solution when there are more hotspot areas but a fixed flight duration. We will also address the challenge of new hotspots appearing and old ones disappearing while the UAV is completing its current mission. Lastly, we will investigate coupling at the word scale in future studies.

## Figures and Tables

**Table 1:** Variables Description.

Symbol	Meaning
U	Ground users (GUs)
*N*	Number of hotspots
*T*	Battery life time
$l_{0}$	UAV’s initial location
$l_{T}$	UAV’s final location
$q_{u}\left(t\right)$	UAV’s trajectory
${\bar{q}}_{u}$	Sequence of hotspots served by the UAV
$h_{n}$	*n*th hotspot served by the UAV
$N^{\prime }$	Total number of hotspots served along the trajectory
L	Set of possible trajectories to follow by the UAV
$\mathrm{Pr}(h_{n+1}|h_{n},\tau_{n+1})$	Probability to move toward hotspot $h_{n+1}$ after visiting $h_{n}$ at time $T-\tau_{h_{n}}$
$\tau_{h_{n}}$	Remaining time to go back to the original location after serving $h_{n}$
N	The set of available hotspot areas
K	The set of GUs distributed across the total geographical area
$K_{n}$	The set of GUs belonging to the *n*th hotspot
$p_{k_{n}}=\left[x_{k_{n}},y_{k_{n}}\right]$	The coordinate of GU $k_{n}$ belonging to the $K_{n}$
$p_{n}=\left[x_{n},y_{n}\right]$	Center of *n*th hotspot
$r_{n}$	Radius of the *n*th hotspot
R	The set of the average data rate of all the available hotspots
$R_{n}$	Data rate of the *n*th hotspot
*t*	Time slot
*u*	UAV
$g_{k_{n},u}\left(t\right)$	Channel gain between GU ($k_{n}$) and UAV (*u*)
K	Channel factor
$f_{c}$	Carrier frequency
*c*	Speed of light
α	Path loss exponent
${\mathrm{Pr}}_{\mathrm{LoS}}$	Probability of line-of-sight
${\mathrm{Pr}}_{\mathrm{NLoS}}$	Probability of non-line-of-sight
$\mu_{LoS}$	Additional attenuation for line-of-sight links
$\mu_{NLoS}$	Additional attenuation for non-line-of-sight links
$d_{k_{n},u}\left(t\right)$	Distance between GU $k_{n}$ and UAV *u* at time *t*
$r_{K_{n}}$	Achievable data rate in hotspot *n*
$B_{k_{n}}$	The bandwidth of the resource block (RB) allocated to user $k_{n}$
$p_{k_{n}}$	Transmit power of user $k_{n}$
$\sigma^{2}$	Power spectral density of the additive white Gaussian noise
D	Training set of realizations representing *M* examples
$L^{†}$	Set of the sequences of hotspots selected by TSPWP to solve *M* examples
$Q^{†}$	Set of trajectory instances generated by TSPWP
S	Set of clusters generated by GNG
${\tilde{l}}_{m}$	Generalized letter
$A_{{\tilde{l}}_{m}}$	Adjacency matrix
$A_{\tilde{l}}$	Global adjacency matrix
$\Pi_{\tilde{l}}$	Global transition matrix
D	Degree matrix
$\Theta_{e_{m}}$	Tokens
$\Pi_{\Theta }$	Tokens transition matrix
$w_{T,e_{m}}^{o}$	Words on order
$w_{T,e_{m}}^{p}$	Words on motion
$w_{T,e_{m}}^{c}$	Coupling word

**Table 2:** Simulation Parameters.

Parameter	Value	Parameter	Value
$P_{u}$	1 W	α	2
$B_{RB}$	180 KHz	$\sigma^{2}$	−104 dBm
$\mu_{Los}$	3	$\mu_{NLos}$	23
*N*	80	*M*	1000

## Data Availability

Not applicable.

## Abbreviations

The following abbreviations are used in this manuscript:

UAV	Unmanned aerial vehicle
LoS	Line of sight
NOMA	Non-orthogonal multiple access
GPS	Global positioning system
IoT	Internet of things
AI	Artificial intelligence
ML	Machine learning
RL	Reinforcement learning
TSPWP	Travelling salesman problem with profits
GDBN	Generalized dynamic Bayesian network
C-MGDBN	Coupled multi-scale generalized dynamic Bayesian network
DP	Dynamic programming
WSN	Wireless sensor node
MILP	Mixed integer linear programming
TSP	Travelling salesman problem
GA	Genetic algorithm
PSO	Particle swarm optimization
ACO	Ant colony optimization
QoE	Quality of experience
QL	Q-learning
DQL	Deep Q-learning
FBS	Flying base station
GU	Ground users
RB	Resource block
OFDMA	Orthogonal frequency division multiple access
NLoS	Non-line-of-sight
AWGN	Additive White Gaussian Noise
C-GDBN	Coupled Generalized dynamic Bayesian network
M-GDBN	Multi-scale generalized dynamic Bayesian network
GNG	Growing neural gas
POMDP	Partially observable Markov decision process
KF	Kalman filter
PF	Particle filter
